# New insights on lake sediment DNA from the catchment: importance of taphonomic and analytical issues on the record quality

**DOI:** 10.1038/s41598-019-50339-1

**Published:** 2019-10-11

**Authors:** C. Giguet-Covex, G. F. Ficetola, K. Walsh, J. Poulenard, M. Bajard, L. Fouinat, P. Sabatier, L. Gielly, E. Messager, A. L. Develle, F. David, P. Taberlet, E. Brisset, F. Guiter, R. Sinet, F. Arnaud

**Affiliations:** 1 BioArch-Department of Archaeology, University of York, York, YO10 5DD UK; 2 EDYTEM, UMR 5204 CNRS, Univ. Savoie Mont Blanc, Pôle Montagne, 73376 Le Bourget du Lac, France; 3 Univ. Grenoble Alpes, Univ. Savoie Mont Blanc, CNRS, LECA, 38000 Grenoble, France; 4 Department of Environmental Science and Policy, Università degli Studi di Milano, Via Celoria 26, 20133 Milano, Italy; 5 CEREGE, UMR CNRS 7330, IRD 161-Marseille Université, Technopôle de l’Arbois Méditerranée, BP 80, 13545 Aix en Provence cedex 4, France; 6 Aix-Marseille Univ, Avignon Univ, CNRS, IRD, IMBE, Aix-en-Provence, France; 7 Institut Català de Paleoecologia Humana i Evolució Social (IPHES), Tarragona, Spain; 8 Àrea de Prehistòria, Universitat Rovira i Virgili, Tarragona, Spain

**Keywords:** Molecular ecology, Palaeoecology, Limnology

## Abstract

Over the last decade, an increasing number of studies have used lake sediment DNA to trace past landscape changes, agricultural activities or human presence. However, the processes responsible for lake sediment formation and sediment properties might affect DNA records via taphonomic and analytical processes. It is crucial to understand these processes to ensure reliable interpretations for “palaeo” studies. Here, we combined plant and mammal DNA metabarcoding analyses with sedimentological and geochemical analyses from three lake-catchment systems that are characterised by different erosion dynamics. The new insights derived from this approach elucidate and assess issues relating to DNA sources and transfer processes. The sources of eroded materials strongly affect the “catchment-DNA” concentration in the sediments. For instance, erosion of upper organic and organo-mineral soil horizons provides a higher amount of plant DNA in lake sediments than deep horizons, bare soils or glacial flours. Moreover, high erosion rates, along with a well-developed hydrographic network, are proposed as factors positively affecting the representation of the catchment flora. The development of open and agricultural landscapes, which favour the erosion, could thus bias the reconstructed landscape trajectory but help the record of these human activities. Regarding domestic animals, pastoral practices and animal behaviour might affect their DNA record because they control the type of source of DNA (“point” *vs*. “diffuse”).

## Introduction

### History and potential of the lake sediment DNA (sedDNA)

The earliest studies of ancient DNA (aDNA) from lake sediment archives (lake sedDNA) date to the late-1990s^1^. However, it is only during the last eight years there has been the extensive application of molecular biological techniques in lake sediment analyses, with a significant contribution over recent years^2–7^. Lake sediments accumulate through time, as both autochthonous (in-lake biological production and chemical precipitation) and allochthonous (particles brought from the catchment and beyond) materials, which can contain DNA, and are preserved within lacustrine environments. The study of such sediments using molecular biological techniques has enormous potential for the identification of organisms present within the “lake’s sediment source area” (i.e., the lake itself, its catchment area as well as the atmosphere) and thus to trace changes of biodiversity over time, from the scale of the population to that of the ecosystem. This approach allows us to address a wide range of questions, especially in ecology and about the nature of human-environment interactions over time^4,8,9^. Before 2008, there had been relatively few studies of lake sedDNA from terrestrial organisms, with all of these studies focussing on pollen DNA^10,11^. On the contrary, many studies have focused on aquatic organisms^1,12–18^. This may be due to the perception that the DNA from organisms within the lake would be accumulated in higher quantities in the sediments compared to the DNA derived from the catchment area. However, since 2008, researchers have successfully tracked organisms derived from terrestrial environments, using bulk sediments and focusing on the analysis of extracellular or total DNA^19^. These studies on bulk sediments targeted plants^20–29^, mammals^24,30^, humans and/or animal specific faecal bacteria^30–35^, and more recently other eukaryotes, such as fungi and worms^5,36^. The aim of such research includes the reconstruction of past biodiversity, vegetation cover, landscape, and climate changes, the nature of agro-pastoral activities and the relationships between humans and landscapes. Such studies have demonstrated the great potential of this tool in providing new knowledge for palaeoecology and archaeology. However, as discussed below, there have been several studies that have interrogated the reliability and accuracy of lake sedDNA results. To our knowledge, the data and analyses presented here constitute the first study that explicitly engages with a range of pre and post-depositional taphonomic process from a range of very different lake catchments. Here, our aim is not to reconstruct a particular ecological dynamic or environmental history but instead offer a model or framework that others might employ and develop when they plan, execute and interpret lake sedDNA research and data. While this pilot study is only based on three lakes, it represents a first step and is a timely contribution for a community increasingly using lake sedDNA.

### Issues and limits: taphonomic and analytical considerations

#### Plant DNA records

**Figure 1:**
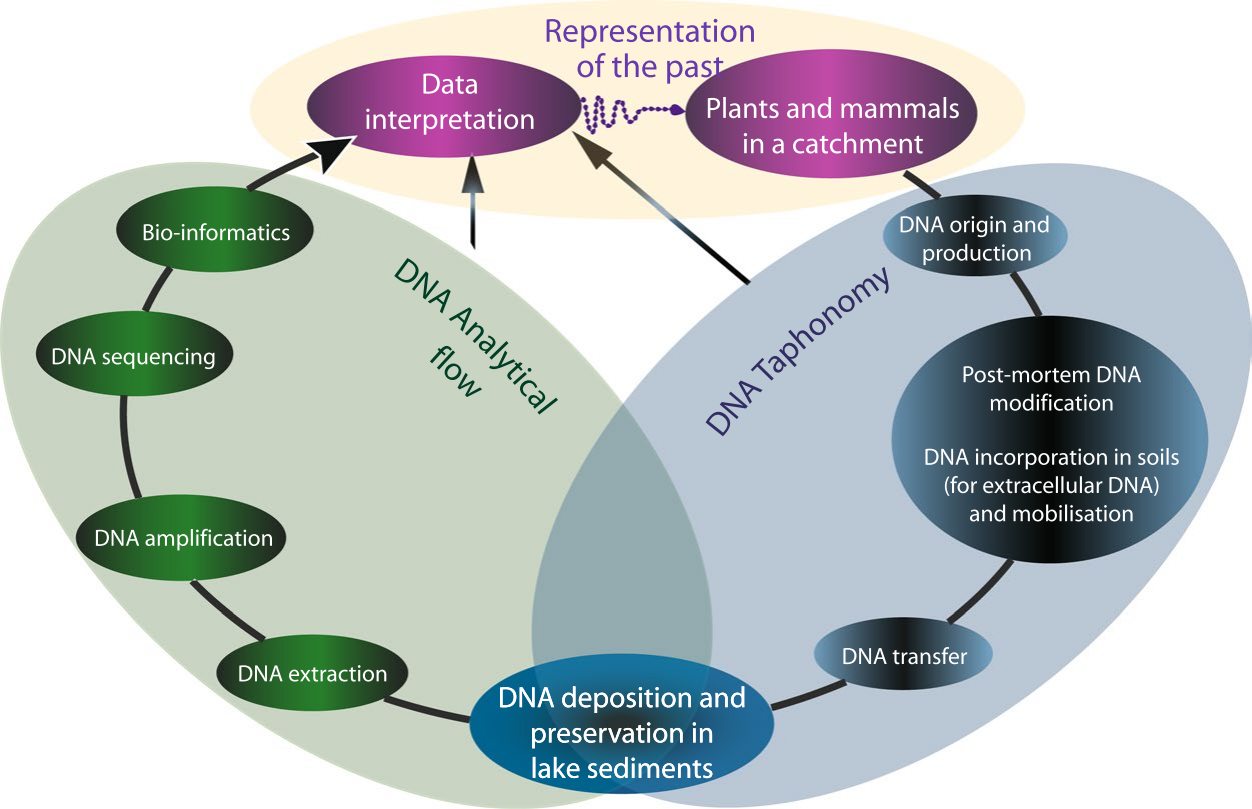
Flow chart of taphonomic and analytical processes likely to affect reconstructions of the past, especially reconstructions of landscapes and agricultural activities.

Along with research that highlights the reliability of lake sedDNA in tracking vegetation changes, many studies have questioned the interpretation of some data, raising concerns over analytical and/or taphonomic processes (modified from^37^; Fig. 1). Taphonomic processes refer to all processes that govern the production, transfer, incorporation, and preservation of the lake sedDNA. For instance, Pedersen *et al*.^38^ did not detect a substantial proportion of DNA from the local flora, which was independently identified by macrofossils. They proposed multiple, non-exclusive explanations, such as the high abundance of some taxa that may overwhelm the rarest taxa. The taxonomic resolution and assignment rate could have been limited by the degradation of DNA sequences, the sequencing depth or the incompleteness of the reference database. Indeed, in a more recent study, also from the Arctic, the authors obtained superior taxonomic recovery between data from DNA and macrofossils analyses, probably due to the use of an almost complete reference library, as well as optimised extraction protocols (sediment quantity) and sequencing conditions^20^. Several studies also revealed discrepancies between records of plant DNA, pollen and macrofossils, which may reflect differences in the source (production, origin), modes of transfer, and preservation conditions for these different vegetation-cover proxies^22,28,38^. Whereas taphonomic processes are relatively well-understood for pollen and plant macro-remains, their understanding for lake sedDNA is still limited^2,8,37^, especially for extracellular DNA, which by definition excludes the DNA from pollen and plant macro-remains. However, a recent review^2,19^, along with two studies that compared modern vegetation with pollen and DNA analyses from surface sediments from a large set of lakes in different vegetation environments (tundra to forest-tundra environments^39^; boreal and alpine^40^) have suggested the following: (1) pollen does not significantly contribute to DNA records, (2) the DNA has a local origin and probably has a similar source as the macrofossils, (3) aquatic plants are well-represented, (4) taxa detection seems to depend on the distance to lakeshore, the relief and its abundance (biomass) in the vegetation, (5) different physical and chemical sediment characteristics might have an impact on the DNA preservation. These studies targeted both intra and extracellular DNA, of which the respective contribution to the sediments remains unclear^2^ while the taphonomic processes affecting each of these DNA pools can be expected to differ.

#### Mammal DNA records

Some studies have also raised questions regarding the taphonomic processes that affect the mammal DNA record. For example, Giguet-Covex and co-authors^24^ did not find sheep extracellular DNA in modern sediments from a small subalpine lake (Lake Anterne, 2063 m a.s.l, Northern French Alps), despite the fact that sheep flocks are present today in the catchment. Here, low stocking-rates and scattered distributions of domestic animals, triggering a low biomass and a “diffuse source” of DNA, have been proposed as an explanation for the non-detection of DNA. On the contrary, high stocking-rates and/or the existence of areas used for the herding or flocking of animals (e.g. enclosures or folds), which represent a “point source” of DNA because of the « concentration effect » of animals, might explain the enhanced supply of mammal DNA in the sediments during earlier periods^24^. Moreover, urine and faeces, the two primary sources of animal DNA^41,42^ - are produced mainly during the night within the enclosures or folds^43^. The presence of enclosures within a catchment is thus expected to significantly favour the detection of domestic animal DNA. Another study that aimed to identify the presence of humans in a catchment using human-specific bacteria DNA also proposed potential biases in the record due to taphonomic issues^31^. In fact, the absence of human-specific bacteria DNA from a core where the pollen data suggests the presence of humans might be explained by DNA concentrations that were below detection limits. For example, situations where human camps/villages are at some distance from the lake or the inlet are expected to limit the DNA transfer to the lake. Moreover, situations when the population density is low are expected to limit the DNA production and biomass. An alternative explanation might also be that pollen reflects human activity from the wider region.

#### “Time shifts”

Several studies have raised the issue of potential “time shifts” in lake sediment DNA records, due to DNA leaching through sediment layers^28^ or DNA storage in soils and its delayed release into the environment several centuries after its production^24^. The delayed release into the environment of molecules stored in soils for several decades has already been observed for pesticides, which are persistent molecules^44^. In a study of alpine soils, it was demonstrated that DNA from crops cultivated over 50 years ago could be detected^45^. This study also shows a significant correlation between the proportion of DNA in soils and the proportion of above ground biomass for different functional plant groups, suggesting that the DNA transported by soil erosion will primarily reflect the ecosystem established at the time of the erosion event, and the influence of older DNA on the overall DNA signal will be minimal. This is supported by recent studies in which DNA accurately recorded the timing of changes in a vegetation cover and mammal distribution, in accordance with detailed evidence from historical and other sedimentological sources^28,29^. This high level of concordance with an independent approach highlights not only the low levels of older DNA stored in soils but also suggests limited DNA leaching through the sediment layers.

#### DNA degradation/preservation processes

DNA degradation and preservation processes within the lake water-column and sediments also have to be considered. DNA preservation and degradation are the most extensively studied taphonomic processes, as these issues are important for several research communities. These communities are interested in nutrient cycles, gene transfer, palaeoenvironmental reconstructions, or genetic studies from archaeological remains such as bones. DNA degradation is triggered by both abiotic and biotic mechanisms. 

From the moment of cell death, DNA repair mechanisms cease, and DNA starts to degrade via several chemical reactions (oxidation, hydrolysis, alkylation and Maillard reaction). These processes act both inside and outside of the cells after their lysis, thus affecting both intracellular and extracellular DNA^46,47^. The rate of chemically-induced degradation is controlled by several environmental factors. Low temperature, high salt concentration (high ionic strength) and high pH, limit the hydrolysis and thus favour DNA preservation^48–50^. Environments protected from ultraviolet (UV) radiation also favour DNA preservation as this radiation causes DNA damage^50^. The extracellular DNA is also affected by microbial activity. In fact, the degradation by DNases produced by bacteria is considered the primary mechanism responsible for extracellular DNA degradation^51^. However, DNA can be protected from this process when it is adsorbed onto charged surfaces (clays and humic substances), or absorbed into the crystal lattice of fine particles, amorphous crystals and particulate organic compounds^51,52^. This protection can also result from the inactivation of DNases via their binding onto particles^53^. The binding of extracellular DNA onto particles, as well as the degree of protection, are complex processes as they are dependent on; the mineralogy of the sorbent, the presence of organic material, pH conditions, and the ionic strength and length of the DNA molecules^54,55^. In soils, nucleic acids released from cells bind quickly to particles^51,53,55,56^, which delays DNA degradation and might explain the detection of a few sequences of crop DNA in the alpine soils 50 years after arable agriculture had come to an end^45^. Inside the lake, bacterial activity, oxygenation, salt concentration, organic and mineral particles, UV penetration and pH conditions can vary through time and thus differentially affect the DNA preservation. When sediments are deposited in the lake bottom, they quickly become anoxic after burial, which limits microbial activity and thus favours long-term DNA preservation. However, the uppermost sediments often represent an active layer that can significantly modify the concentration and composition of DNA^7^. With burial, DNA is entirely protected from UV radiation. In marine sediments, it has also been shown that a high proportion of extracellular DNA is bound to minerals or humic substances^56–58^. Given the mechanisms of DNA protection afforded by binding, the absence of oxygen and UV radiation, aquatic sediments are, *a priori*, suitable environments for DNA preservation^19^. However, low bacterial activity, along with the binding of DNA on particles, does not prevent chemically induced DNA degradation, especially hydrolysis. DNA degradation should result in a decrease of the DNA pool with time, and a decrease in the size of DNA fragments still present within the sediment. A time-dependent DNA decrease was reported in a study of dinoflagellate DNA from fjord sediments in Antarctica^59^, and several studies reported the loss of long fragments with age^4,27,60^. Ageing also triggers cytosine to thymine substitutions at the single-stranded ends of the DNA fragments, which can be used to discriminate between ancient DNA sequences and contaminations from modern DNA^27,60,61^. DNA preservation can also vary among different groups of organisms as well as among different species of the same group^59,62–64^.

### Challenges ahead and aim of the study

In the light of the preceding discussion and the issues raised therein, it is apparent that there is a need to investigate the potential distortions within lake sediment DNA records due to the taphonomic processes (production, transfer, preservation of DNA) that affect DNA in sediments. Then, we are obliged to consider biases or issues with our analytical procedures (extraction/amplification/identification)^9,8,37^ (Fig. 1). Without a good understanding of these potential biases, the potential of lake sediment DNA analyses cannot be fully realised. Of particular importance is the issue as to whether the DNA archived within sediment represents a reliable diachronic signal. More specifically, we have to ask if the following characteristics or processes are constant over time: 1) the source of DNA, 2) the processes and efficiency of DNA transfer, and 3) the preservation conditions of DNA?

**Figure 2:**
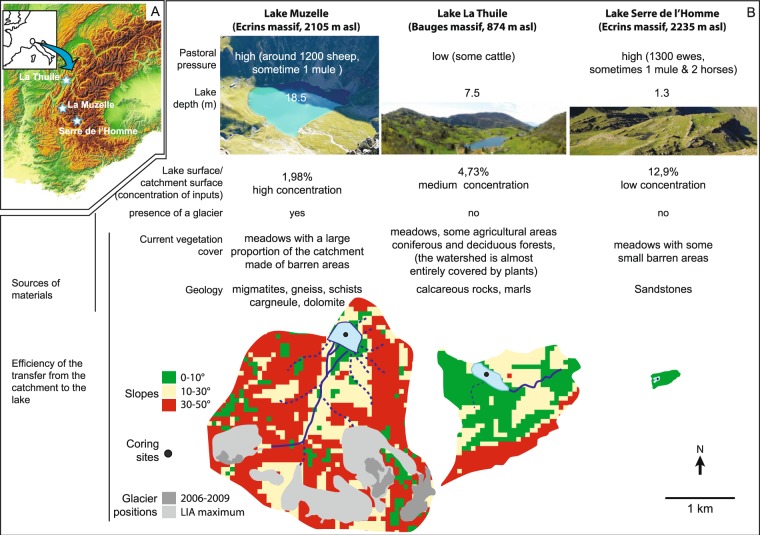
Presentation of the study sites. (**A**) Location of sites (map background purchased, along with copyright, from Map Resources [Extended Use Electronic/Broadcast License]). (**B**) Presentation of the characteristics of each catchment-lake system (pastoral pressure, physical characteristics and plant cover).

Our review of the literature demonstrates that our understanding of DNA preservation processes is improving. However, few studies have focused on identifying terrestrial DNA sources and transfer processes from catchments to lakes. In order to engage with all of these processes and questions, we present the empirical analysis of temporal lake sedDNA datasets from three mountainous lake-catchment systems characterised by a range of erosion dynamics that are the product of different geological formations, topographical characteristics and vegetation and soil cover characteristics (Fig. 2A,B). Our review of these different contexts and concomitant processes allows us to elucidate complex, catchment specific, taphonomic (i.e. source and transfer) processes. Both plant and mammal extracellular DNA were investigated using the DNA *metabarcoding* approach, which is the amplification and sequencing of DNA molecules found in the environment using universal markers^65^. This extracellular DNA may represent the main DNA pool in sediments^57,66^ and is of great interest as it may provide the most integrated view of aquatic, sedimentary and terrestrial biodiversity^58^. Here, we only focused on this particular DNA pool to avoid the extraction of DNA from plant macro-remains, which might lead to an overrepresentation of these taxa and limit the detection of the other, rarer taxa. Sedimentological and geochemical data were also acquired, and provide information on the processes of sediment production, transfer, and deposit, as well as of lake water physicochemical conditions. Pollen or coprophilous fungi data were included in the study as complementary evidence of vegetation cover changes and domestic herd presence. All these data are essential for our understanding of the processes that drive the production and preservation of DNA records. In addition, these data allow us to consider how changes in taphonomic conditions over time can affect the quality of the DNA record and thus facilitate the evaluation of our landscape and land-use reconstructions.

## Results and Interpretations

### Plant DNA detected in the three lakes

The analysis of sequencing data was realised using appropriate bioinformatic tools (OBITOOLS software) and a filtering procedure based on the percentage of similarity with sequences in the reference database, the identification of potential contaminations and exotic taxa, and the consideration of stochastic detections. The detailed procedure is described in the “Materials and methods” section and is discussed in the Supplementary materials section 2.2. After this filtering procedure, 107 and 83 MOTUs (Molecular Operational Taxonomic Units) of plants were detected in lakes La Thuile and Muzelle, respectively, while only 19 MOTUs were found in Lake Serre de l’Homme. In Lake Muzelle, we exclusively detected DNA from terrestrial plants (100% of the reads; a read is a sequence of base pairs corresponding to a single DNA fragment and produced by a sequencer). Lake La Thuile presents a mixed recording, but most of the DNA reads are of terrestrial origin (71% of reads distributed in 96 MOTUs, Table 1). Conversely, most of the DNA reads detected in Lake Serre de l’Homme are aquatic in origin (79% of reads distributed in 7 MOTUs but probably only representing 3 different taxa, Table 1 and Supplementary Fig. 3).

**Table 1:** Synthesis of plant DNA results for the three lakes.

Lakes			*La Thuile*	*Muzelle*	*Serre de l’Homme*
*number of samples*			50	30	41
*replicate number performed*			4	4	8
*Illumina Hi-seq run numero*			run 1	run 1	run 2
number of MOTU	*Terrestrial*		96	83	12
	*Aquatic*		11	0	7
number of reads	*Terrestrial*		796266	1836110	1205395
	*Aquatic*		326988	0	4517931
	*Terrestrial (%)*		70,90	100	21,10
	*Aquatic (%)*		29,10	0	78,90
*% of samples with x positive replicates*	*Terrestrial*	***0***	2	0	**58,5**
		***1***	4	0,3	**26,8**
		***2***	6	0	4,9
		***3***	**12**	**10**	9,8
		***4***	**76**	**86,7**	0
		***>4***	No analyses	No analyses	0
	*Aquatic*	***0***	28	0	44
		***1***	22	0	22
		***2***	2	0	22
		***3***	14	0	0
		***4***	34	0	0
		***>4***	No analyses	No analyses	**12**

La Thuile and Muzelle were analysed in the same sequencing run.

**Figure 3:**
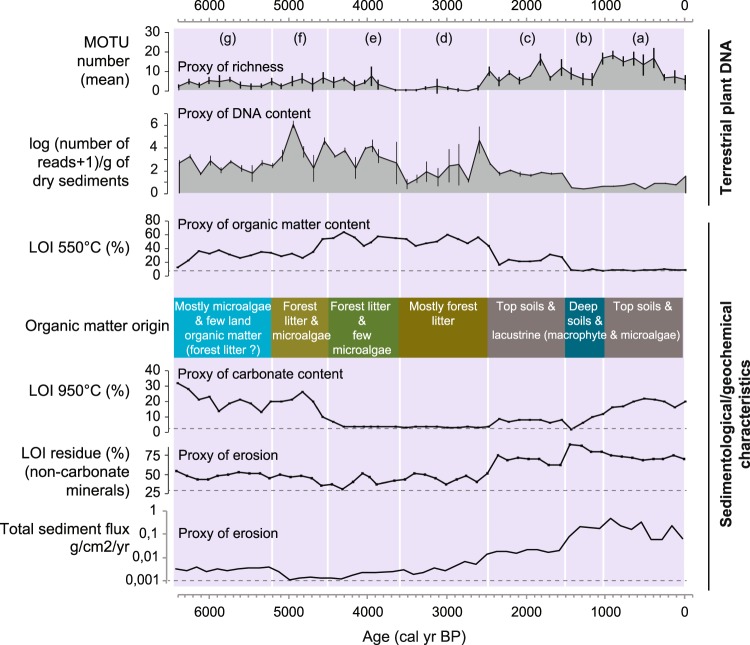
Comparison between global terrestrial plant DNA and the sedimentological/geochemical properties of sediments in Lake La Thuile over the last 6500 years. To study the behaviour of land plant extracellular DNA we focused on the proxies of the richness (mean and standard deviations of the number of MOTU) and the DNA contents in the samples (mean and standard deviations of the log(number of DNA reads + 1)/dry mass of sediment). These variables were compared to several selected sedimentological and geochemical data: the organic matter content (LOI_550 °C_) and origin, the non-carbonate mineral matter (LOI residue), the carbonates (LOI_950 °C_) and the total sediment flux (g/cm^2^/yr). The organic matter origin is determined from the combination of data from pyrolysis Rock Eval analyses (Hydrogen Index in mg HC/g TOC and Oxygen Index in mg O_2_/g TOC^69^), X-Ray fluorescence core scanner analyses (Si/Ti as a proxy of biogenic silica^67^), the lithological description and the aquatic plant DNA analyses (Supplementary materials, Figs 2 and 4). Seven specific phases of changes in DNA content were defined and discussed in the text (purple shaded areas **a–g**). They correspond to different sedimentological and geochemical characteristics, which inform hypotheses explaining the behaviour of the extracellular DNA from the catchment.

Based on the comparison between the proportions of samples in which terrestrial plants are detected in 0, 1, 2, 3 or 4 replicates, it is clear that the low terrestrial plant richness detected in Lake Serre de l’Homme also corresponds to very low quantities of DNA extracted from the samples compared to the two other lakes. In fact, terrestrial plants were never detected in more than three replicates over eight. In addition, in 85% of the samples, either terrestrial plants were not detected, or they were only detected in one replicate over eight (Table 1). However, in 12% of the samples, aquatic plants were detected in more than four replicates (34% of the samples contain aquatic plant DNA in more than one replicate). Conversely to Serre de l’Homme, terrestrial plant DNA was detected in all four of the replicates in most of the samples from lakes Muzelle and La Thuile (87% and 76%, respectively) (Table 1). Some samples from Lake La Thuile also lacked plant DNA (Table 1, Fig. 3). The three lake-catchment systems are thus characterised by different plant DNA records in terms of quality. The absence of terrestrial plant DNA in most samples from Serre de l’Homme, and in some samples from La Thuile, clearly raises the question of taphonomic issues. In order to assess the potential existence of other taphonomic biases and understand their origin, it is necessary to study each record in detail, integrating other proxies for vegetation cover (pollen data) and data that characterises sedimentary dynamics (sedimentological and geochemical data).

#### La Thuile: evidence for the roles of erosion processes and vegetation cover in the production of the DNA record

**Figure 4:**
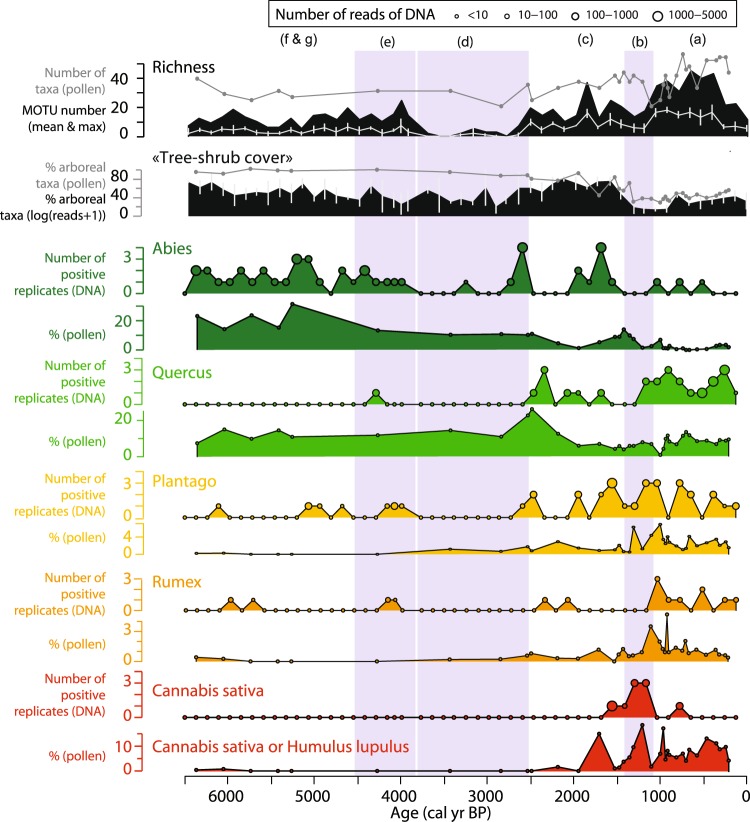
Community composition of terrestrial plants provided by the DNA and pollen analyses from Lake La Thuile. The temporal evolution of the richness, the percentage of arboreal taxa, and some taxa are presented for both methods. The taxa presented here were selected in order to show examples of temporally consistent and inconsistent records, which can have different explanations. The other taxa are presented in Supplementary Fig. 8. For the richness and the percentage of arboreal taxa determined from the terrestrial plant DNA dataset, we present the mean values and standard deviations of the four replicates. The maximum richness, i.e. cumulating all the replicates is also presented as it provides a more pertinent absolute value to compare with that of pollen. For each taxon in DNA, the size of circles is proportional to the number of reads (see scale on the top of the figure). The purple shaded areas underline the periods (**b,d,e**), when no or very few DNA was detected.

The diachronic record of terrestrial plant DNA concentration (Fig. 3) can be divided into seven phases ((a) from 0 to 1000 cal. BP, (b) from 1000 to 1400 cal. BP, (c) from 1400 to 2500 cal. BP, (d) from 2500 to 3600 cal. BP, phase (e) 3600 to 4500 cal. BP, (f) from 4500 to 5200 cal. BP and (g) from 5200 to 6400 cal. BP). These phases correspond to changes in environmental conditions inferred from the sedimentological and geochemical proxies^67^. In most of these phases (a, b, c, e and g), the terrestrial plant DNA concentration is positively correlated with the organic matter content (Pearson correlation coefficient r = 0.82, p < 0.001 excluding phases d and f; Fig. 3). This relationship probably reflects the significant role of biomass production that has been described in previous studies^2,40^. However, phases (d) and (f) do not follow this pattern. They are, respectively, impoverished and enriched in DNA, compared to the organic content. Phase (d) is also characterised by very low carbonate content (<4%) (Fig. 3), which might indicate acidic conditions in the water column. Acidic conditions are not favourable for DNA preservation^48,49,50^. Moreover, our method of DNA extraction might not be efficient enough to unbind organically (humic substances)-complexed DNA^58^, which might be an important pool of extracellular DNA in this part of the sedimentary sequence mostly made of leaves and needles^67^. Humic substances are also known to inhibit the PCR reaction^68^. The low-DNA content in phase (d) might thus be due to unfavourable preservation conditions and/or analytical limits. Phase (f) contains as much organic matter as phase (g), but the DNA content is higher. However, phase (f) contains much more organic matter of terrestrial origin (vs aquatic; cf Fig. 3), derived from the erosion of forest litter and/or the direct input of the upper parts of plants within the lake^67^. Very high levels of organic matter derived from forest litter are also recorded in phase (e), but the DNA content does not significantly increase relative to phase (f). This result is probably due to the presence of humic substances and the acidic conditions suggested by the low carbonate content, as seen in phase (d). Phase (b) has a slightly lower DNA content than phase (a), while organic matter values are similar in these phases. Moreover, this phase presents a very low number of taxa, especially compared to those detected by pollen analyses (Fig. 4). However, this phase is dominated by inputs of material from deep soils, i.e. mineral soil horizons, while phase (a) is dominated by inputs from the soil surface, i.e. organo-mineral soil horizons (Fig. 3^69^). Consequently, sediments are enriched in terrestrial plant DNA when soil-surface horizons (litter and organo-mineral horizons) are transported into the lake. However, this enrichment is lower or non-existent if the lake water is acidic and/or contains humic substances; conditions that do not favour DNA preservation/recovery. On the contrary, the sediments are impoverished in terrestrial plant DNA when the erosion strongly affects the deep soil horizons. This pattern is also evidenced by a PCA analysis (see Supplementary Fig. 8). Consequently, the erosion processes (e.g. sheet erosion, gully erosion or bank undercutting), controlling the origin of the organic matter, are key processes driving terrestrial plant DNA concentration in sediments.

A comparison of the pollen and DNA records allows us to identify other potential taphonomic processes and thereby assess the ways in which these affect the vegetation cover reconstructions derived from the DNA analysis. We consider the floristic richness, the proportion of trees and shrubs, and the 34 taxa detected by both DNA and pollen analyses (sometimes associated at different taxonomic resolution; Fig. 4 and Supplementary Fig. 9). Any temporal differences between the proxies are considered a direct consequence of taphonomic biases.

Both pollen and DNA records show an increase in floristic richness from 2500 cal. BP, i.e. from phase (c) (Fig. 4). However, the results from the DNA analyses reveal a more significant increase (especially in phase (a)). In fact, before 2500 cal. BP, 31 and 11 taxa on average are detected by pollen and molecular analyses, respectively (without taking into account phases d and e of lower DNA detection). From 2500 cal. BP, the number of taxa detected with pollen increases to 34 on average for phase (c) and to 38 for phase (a), whereas with the molecular analyses, the mean number of taxa in phases (c) and (a) reach 19 and 30 on average, respectively. Therefore, the effectiveness of plant-community detection via the DNA analyses may well be higher after 2500 cal. BP. Moreover, from 2500 to 1400 cal. BP (i.e. in phase (c)), an increase of the proportion of arboreal taxa is recorded by DNA whereas pollen data suggest deforestation. The significant increase of the erosion from 2500 cal. BP (see LOI residue and total flux of sediments in Fig. 3^67,69^), supports the argument that deforestation occurred during this phase; a possible consequence of soil instability due to human activity. Consequently, the higher detection of trees (for instance, *Quercus sp*., *Acer sp*., Betulaceae, Ulmaceae and to a lesser extent *Viburnum opulus* and *lantana*, Fig. 4 and Supplementary Fig. 9) and the increase in species richness revealed via the DNA dataset when compared with the pollen data might be explained by the higher erosion rate. In fact, erosion increases the degree of connectivity across the catchment area (i.e. creates new connexions between patches of the catchment and the hydrographic web, including the lake). On the contrary, before 2500 cal. BP, in the forested landscape, there is a probable bias towards the recording of plants that were growing on the lakeshore and the riverside, as suggested by the dominance riparian *Alnus sp*., (*Alnus glutinosa* and *incana*), and by the presence of *Frangula sp*. (Supplementary Fig. 9). This would be due to the transfer of proximal litter or tree leaves into the sedimentary system.

Temporal inconsistencies are recorded between *Cannabis sativa*, detected via DNA analyses, and *Cannabis sativa* or *Humulus lupulus* (from the Cannabaceae family), detected via pollen analyses (Fig. 4). The high values of these pollen, comprising 10–15% of total pollen, suggest that they originate from retting activity. In this case, both pollen and DNA are directly transferred to the lake. Consequently, high quantities of DNA from *Cannabis sativa* can be transferred to the sediments and this might explain the high detection levels during phase (b), i.e. when erosion affects the deep soil horizons and dilutes DNA inputs from other terrestrial plants (Figs 3 and 4). Conversely, in phases (a) and (c), when the erosion predominantly affects soil surface horizons, the DNA from *Cannabis sativa* may be diluted by the DNA from other plants in the catchment. As the DNA from this species becomes rarer, it competes with other more abundant DNA fragments and is therefore no longer amplified. Nevertheless, we can see that for many taxa, DNA and pollen signals are the same (excluding phases b and d). Such trends are particularly coherent for tree taxa such as *Taxus sp*., *Tilia sp*., *Abies sp*., *Alnus sp*., *Fagus sp*., *Cupressaceae* (*Juniperus* with pollen) and *Juglandaceae* (*Juglans* with pollen). Herbaceous plants, like *Rumex sp*., *Plantago sp*., *Mentha sp*./ Mentheae, *Helianthemum nummularium* (*Helianthemum* with pollen) and others also record the same history (Fig. 4 and Supplementary Fig. 9).

#### Serre de l’Homme: evidence for the roles of topographic and physicochemical preservation conditions in DNA recording

**Figure 5:**
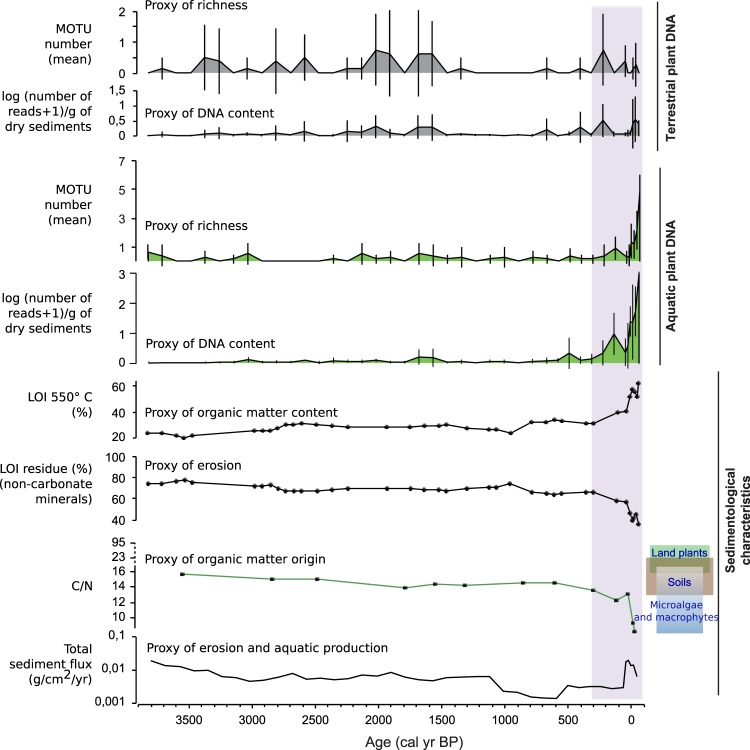
Comparison between plant DNA (terrestrial and aquatic) and the sedimentological/geochemical properties of sediments from Lake Serre de L’Homme over the last 3800 years. To study the behaviour of plant extracellular DNA we focused on the proxies of the richness (mean and standard deviations of the number of MOTU) and the DNA content (mean and standard deviations of the log(number of DNA reads + 1)/dry mass of sediment). These variables were compared to the organic matter content (LOI_550 °C_) and origin (C/N atomic ratio), the content in non-carbonate mineral matter (LOI residue) and the total sediment flux (g/cm2/yr). The ranges of C/N values of land plants (green shaded area), soils (brown shaded area) and algae and aquatic plants (blue shaded area) come from the literature^70–74^. The main change in sediment composition is characterised by an increase in aquatic organic matter accumulation corresponding to an increase in aquatic plant DNA.

Very little terrestrial plant DNA (low DNA concentration and richness) is recorded in Lake Serre de l’Homme (Fig. 5). The sediments mostly comprised non-carbonate mineral matter (35.5–78%) of clastic and biogenic (diatoms) origin as well as organic matter (20.4–62%). The C:N atomic ratio fluctuates from 9.3 to 15.4, i.e. between a pure aquatic end-member and a mixed terrestrial/aquatic end-member^70–74^ (Fig. 5). The sediments contain terrestrial plant macrofossils. The lake catchment is flat, and the “lake surface: catchment surface” ratio is high, which explains the low terrigenous inputs reflected by the low total flux of sediments (between 1 and 20 mg/cm^2^/yr). In these topographical conditions, only the most easily erodible materials are mobilised. These materials may be plant remains that have fallen onto the soils (constituting the source of terrestrial plant macrofossils) as well as the bare soils on sandstones (Fig. 2), which contribute to the non-carbonate mineral matter. These materials are not expected to bear extracellular DNA from plants, which probably contribute to poor detection levels for terrestrial plant DNA. Moreover, poor-DNA preservation conditions may be triggered by soil acidity: pH values of 4.3–5.3 have been recorded on soils developed on the same geological substratum close to the catchment. In addition, poor DNA preservation might also be explained by low water depth that engenders high temperatures and oxygenation at the lake bottom. Higher detection probability of taxa has been demonstrated in studies of deeper lakes in boreal to alpine environments in Northern Norway^40^. In Lake Serre de l’Homme, better in-lake preservation conditions are assumed to have existed from 300–100 cal. BP, due to the higher organic matter production that favoured the establishment of anoxic conditions, thus reducing bacterial activity. These good preservation conditions may contribute to the detection of high quantities of aquatic plant DNA, which is otherwise in agreement with the decrease of the C:N atomic ratio (Fig. 5).

**Figure 6:**
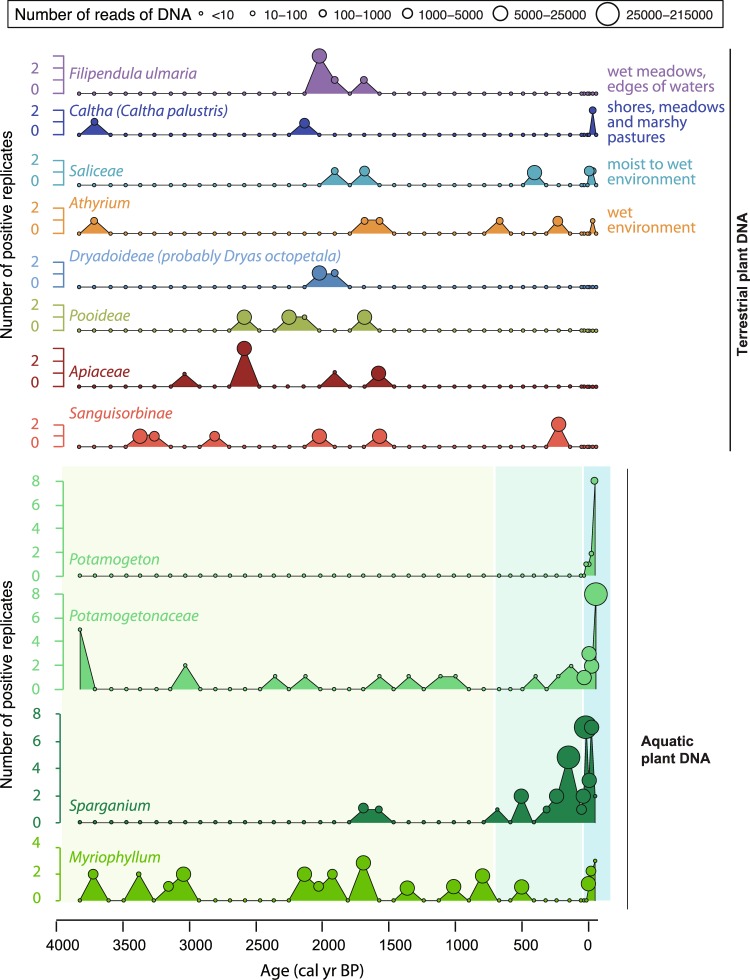
Community composition of terrestrial and aquatic plants provided by the DNA analyses from Lake Serre de L’Homme. For each taxon, the size of circles is proportional to the number of reads (see scale on the top of the figure). Four over eight terrestrial taxa are specific of wet environments. The detection of terrestrial taxa is relatively stochastic and only three taxa are detected in more than one replicate but in one sample (*Filipendula ulmaria*, *Caltha* and *Apiaceae*). However, each aquatic taxon is more frequently detected and often in at least two replicates. Moreover, their detections are clustered in specific periods highlighted by the green areas: the periods 3800–2950 and 2250–700 cal. BP are mostly characterised by *Myriophyllum sp*., the period 700–10 cal. BP by *Sparganium sp*. and the period from 10 to −59 cal. BP the three taxa.

The poor quality of the terrestrial flora reconstruction is characterised by the stochastic detection of only eight different taxa (Fig. 6). At least four of these plants live in wet environments (*Athyrium sp*., *Caltha sp*., *Saliceae* and *Filipendula ulmaria*). The proximity or good connection between these wet environments and the lake might have favoured DNA transfer of plants that grow in these environments, such as DNA from the aquatic plants^28,40^ that appear throughout the Serre de l’Homme record (successions of *Myriophyllum sp*., *Sparganium sp*. and *Potamogeton sp*. as well as *Potamogetonaceae*, Fig. 6). On the contrary, the very poor spatial representativeness of catchment-scale flora at Serre de l’Homme probably reflects the low connectivity between the whole catchment and the lake. This is due to the absence of a well-developed hydrographic network and low erosion, both a consequence of flat topography. The role of catchment relief on catchment flora reconstructions has also been proposed in two recent studies, in Arctic and African environments^40,75^.

#### Muzelle: evidence for the roles of glaciers and the hydrographic web in DNA recording

**Figure 7:**
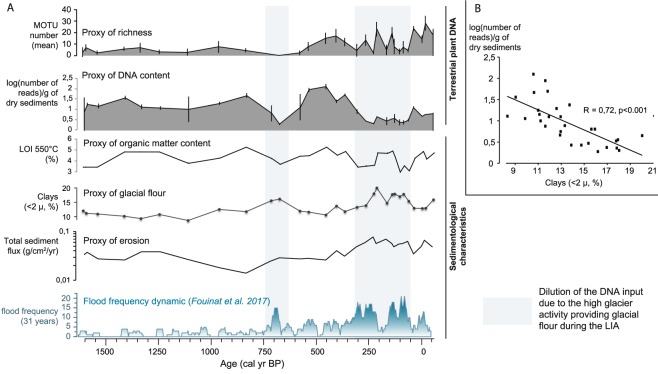
Comparison between terrestrial plants DNA archived in Lake Muzelle sediments and the sedimentological/geochemical properties of sediments. (**A**) Evolutions of the richness (mean values and standard deviations of the four replicates), the contents in DNA reads in the samples (mean number of DNA reads normalised by the dry mass of sediment and standard deviations of the four replicates), the organic matter content (LOI 550 °C), the clay content, the total sediment flux (g/cm2/yr) and the flood frequency over the last 1600 years. Blue areas highlight phases of high inputs of clays and high flood frequency, which corresponds to low DNA concentration in the sediment samples. (**B**) Relationship between the DNA content in the samples and the clay content.

The analyses of the sediments from Lake Muzelle reveal significant variations in terrestrial-plant DNA concentration (from 0.28 to 2.10, Fig. 7A). However, throughout the core, these sediments possess almost homogeneous concentrations of non-carbonate mineral matter (93.6% +/− 0.8) and total organic matter (4.2% +/− 0.6) and carbonates (2.2% +/− 0.4). The sedimentological dynamic of this lake is dominated by significant changes in grain size^76^. The concentration of terrestrial-plant DNA tends to decrease with the increase in clay content (Pearson correlation coefficient r = −0.72, p < 0.0001; Fig. 7B). These inputs of clays increase substantially during two phases; 750–625 and 310–50 cal. BP (Fig. 7A), i.e. during the Little Ice Age (LIA)^77^. In this context, and given the presence of a glacier in the catchment, clays are interpreted as representing a proxy for glacier sediment input (glacial flour) to the lake. In fact, glacier advances triggered by colder and/or wetter conditions produce more glacial flour, which increases the input of clays into the lake, especially during high precipitation events as shown by the increase of the flood frequency^76^. Because these clays do not come from soils covered by plants, no extracellular DNA fragments from terrestrial plants are expected to be bound to these clays. Thus, the inputs of these DNA-free clays might dilute the DNA coming from vegetated-soil erosion and thereby explain the decreases in DNA content when clays increase (Fig. 7A).

**Figure 8:**
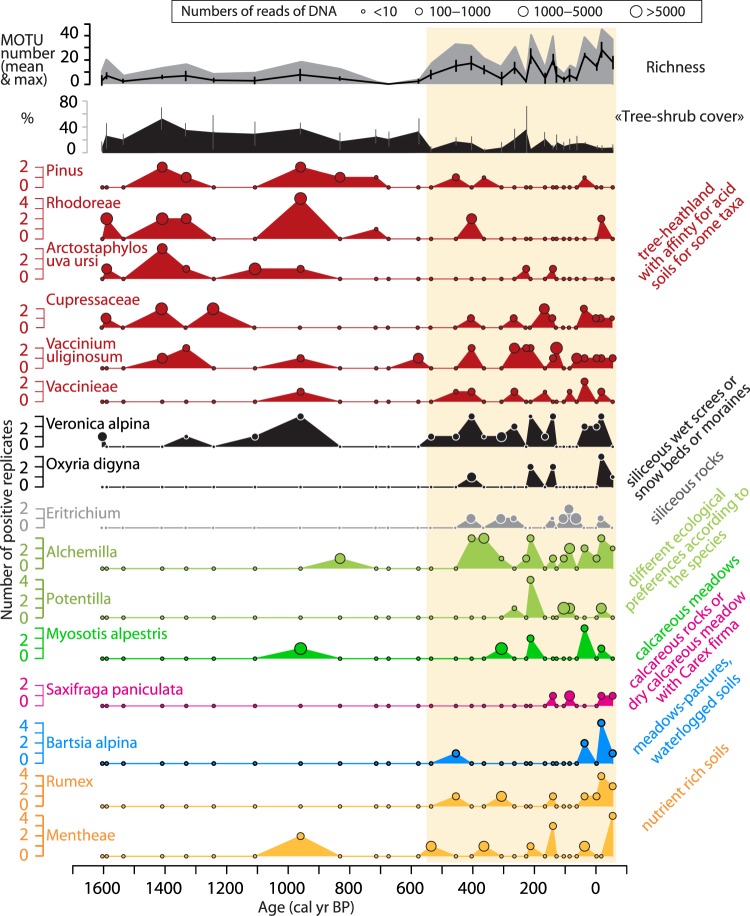
Community composition of terrestrial plants provided by the DNA analyses from Lake Muzelle. The richness (mean and maximum), the percentage of arboreal taxa and several taxa (species and genus) of different ecological preferences (mentioned on the right side of the figure) were selected to document the landscape and environmental changes. *Alchemilla* sp. and *Potentilla* sp. can have different ecological preferences according to the species. However, these pollen types were frequently observed in overgrazed and trampling sites^92^. A study on lake sediments DNA also observed these taxa during phases when pastoral activities with sheep and/or cow were recognised^25^. For each taxon, the size of circles is proportional to the number of reads (see scale on the top of the figure).

The taxonomic richness increases significantly from 550 cal. BP, i.e. when tree-shrub cover % decreases (Fig. 8). From this period, plant communities with different ecological preferences are recorded. In fact, heathland plants, characteristic of well-developed acid soils (e.g. *Vaccinium uliginosum*), are detected together with plants of calcareous meadow (*Myosotis alpestris*), siliceous screes, snow beds or moraines (*Oxyria digyna*, *Veronica alpina*), siliceous rocks (*Eritrichium sp*.), calcareous rocks (*Saxifraga paniculata*), nutrient-rich soils (*Rumex sp*., most of *Mentheae sp*.) and wet environments (*Bartsia alpina*) (Fig. 8). This record of a mosaic landscape may have been favoured by the well-developed hydrographic network connecting different parts of the catchment to the lake (Fig. 2), and the important erosion dynamic as represented by the high total sediment flux (14–77 mg/cm^2^/yr) and by the high contribution of non-carbonate mineral matter (Fig. 7). This mosaic landscape is probably the result of the landscape opening caused by the development of pastoral activities, as suggested by the presence of plants with a preference for nutrient-rich soils. Mammal DNA analyses, considered below, can help test this hypothesis.

### Mammal DNA detection and indirect evidence for pastoral activities

**Figure 9:**
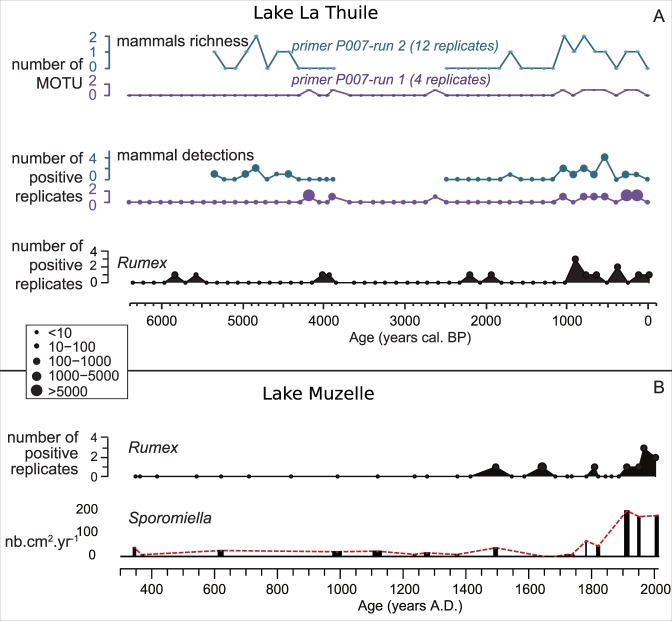
Comparison of proxies of the presence of domestic animals in the aim of studying the taphonomic processes and analytical biases affecting mammal DNA. (A) Comparison for Lake La Thuile between the mammal DNA results obtained from the same primer “mam P007”, but not with the same replicate numbers (4 vs 12). The DNA from *Rumex sp*. is also presented as a proxy of high animal stocking rate or DNA. (**B**) Comparison on Lake Muzelle between the DNA from *Rumex sp*. and spores of coprophilous fungi (*Sporomiella sp*. in Fouinat *et al*.^76^).

Mammal DNA is only detected in the sediments from Lake La Thuile (Table 2), while herds/flocks of domestic animals currently graze on all study sites, with high pastoral pressure around lakes Serre de l’Homme and Muzelle (Fig. 2). In the first run of sequencing (four PCR replicates per sample), only cattle are detected in La Thuile (Table 2), and always in only one replicate. In the second run of sequencing (twelve PCR replicates), the number of positive replicates where mammals were detected increases to four, and we detected two additional taxa (*Ovis sp*. and *Canis sp*. in addition to *Bos sp*.) (Table 2). More mammal DNA is detected from the sediments covering the last thousand years, which correlates nicely with the detection of *Rumex sp*. (Fig. 8A; 9), a nitrophilous plant commonly associated with animal stalls. *Plantago sp*., generally associated with grazing activity because it is resistant to trampling and not eaten by animals (especially *P. alpina* and *P. Lanceolata*), is also detected in previous periods (DNA and pollen, Fig. 4), e.g. from the Late Iron Age to the Early Medieval Period. Its occurrence suggests that herds/flocks of domestic animals might have been present in the catchment during these periods, although the mammal DNA analyses do not reveal the presence of domesticates. This absence of animal DNA before 1000 cal. BP might be due to several factors: (1) a real low number of animals, (2) a dominance of sheep or goats relative to cattle, (3) an absence of areas of animal stalls, (4) an absence of connexion between the area of animal stalls and the hydrographic web, (5) scattered distributions of animals, (6) a predominance of deep-soil horizon erosion between 1400 and 1000 yr cal. BP (Fig. 3), and (7) a combination of these factors. The first two factors lead to low DNA quantity due to low biomass and refer to the notion of Livestock Unit (LU) in agronomy. The third, fourth and fifth factors refer to the notion of stock density (LU/surface unit/time unit) in agronomy. In another alpine lake (Anterne), sheep DNA was detected in only one of eight replicates during the Late Bronze Age, whereas *Plantago sp*. DNA was regularly recorded during this period^24,25^. In this case, the low DNA content may also be explained by a dilution triggered by the significant increase in deep soil-horizon erosion^24,78^. Furthermore, as observed at Lake La Thuile (Fig. 3), this period was also characterised by the detection of few terrestrial plant taxa^26^.

**Table 2:** Synthesis of mammal DNA results from the three lake sediment cores.

Primer	PCR replicate number	Illumina Hi-se run	detected taxa		
			La Thuile	Muzelle	Serre de l’Homme
Mam-P007	4	1	*Bos sp*.	No DNA	No analyses
Mam-P007	12	2	*Bos sp., Ovis sp., (Canis sp.)*	No analyses	No analyses
Mam-P007	8	3	No analyses	No analyses	No DNA

The absence of mammal DNA in sediments from Lake Muzelle is quite unexpected. Indeed, in addition to high pastoral pressure around the lake today, DNA from *Rumex sp*. and spores from coprophilous fungi (*Sporomiella sp*.)^76^ are found in the sediments dated to the last few centuries (Fig. 9B). These results strongly suggest the presence of domestic flocks/herds at least during the recent past. Coprophilous fungi spores, as well as extracellular DNA from both *Rumex sp*. and domestic animals, are supposed to share the same area of production. *Sporomiella* spores mainly come from the faeces of herbivores, mammal DNA is assumed to be largely derived from dung, and urine^42^ and DNA from *Rumex* comes from places of high nutrient accumulation, such as domestic animal stalls where faeces accumulate (hence the good correspondence with the mammal DNA observed for La Thuile). However, the production (and thus concentration) of each of these proxies, as well as their distribution through the soil profiles, can be different. Consequently, the non-detection of mammal DNA in the sediments from Lake Muzelle might be due to low production/concentration of mammal DNA compared to DNA from *Rumex sp*. as well as *Sporomiella sp. spores*. Another explanation might be the different detection limits between these distinct proxies. The difficulty of detecting mammal DNA is well illustrated by the repeated amplification of DNA from sediments from Lake La Thuile. In fact, improved detection (higher numbers of positive replicates and more taxa) of mammal DNA is attained when the number of DNA replicates is increased (Lake La Thuile Table 2 and Fig. 9A), as this increases the detection probability of “rare” taxa^79,80^. In particular, *Ovis sp*. is consistently detected in Lake La Thuile only after the performance of numerous PCR replicates (Table 2). Even if these taxa are not “rare” in the catchment, because of contaminations of samples by human DNA during the lab work (still high even with the use of blocking primers, see Supplementary Fig. 6), these taxa have to be considered as “rare” in our samples. In fact, the DNA sequences from the domestic animals are diluted by the high amount of sequences from the human contamination. Moreover, these human DNA sequences are not degraded and thus preferentially amplified during the PCR. Consequently, the low number of replicates analysed in Lake Muzelle (only four), could contribute to the non-detection of the domestic animals.

The absence of mammal DNA in the sediments from Lake Serre de l’Homme, where spores of *Sporomiella sp*. are also detected and 8 replicates were performed, is probably due to the low detrital input combined with poor-DNA preservation conditions as was hypothesised for terrestrial plants.

## Discussion

**Figure 10:**
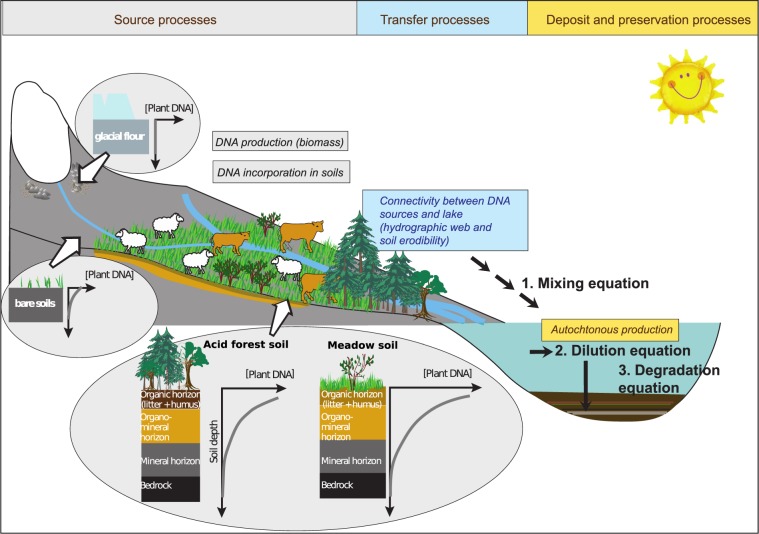
Proposition of a model describing the processes driving the archiving of extracellular DNA from plants and mammals in the lake sediments. Taphonomic processes acting at the source and driving the transfer, deposit and preservation of the DNA in the lake sediments are summarised.

Based on the case studies and the literature review presented above, we propose a model summarising the archiving of the extracellular DNA from the catchment around a lake (Fig. 10). This model can be used to guide the choice of lakes most suitable for the reconstruction of the catchment history (landscape changes, agropastoral activities, biodiversity).

The model integrates three equations. The first is a mixing equation between the different materials affected by erosion in the catchment and transferred to the lake. This equation can be written as follows, for one taxon (Eq. 1) and several taxa (Eq. 2):

1 $$[{{\rm{DNA}}}_{Taxaj,TERRinit}]=\mathop{\sum }\limits_{{\boldsymbol{i}}=0}^{{\boldsymbol{x}}1}[{{\rm{DNA}}}_{Taxaj,Sourcei}][{\rm{Source}}\,i],$$

where [DNA_*Taxa j,TERRinit*_] is the concentration of one taxon “j” in the different terrigenous materials affected by the erosion (log(N reads + 1)/g of terrigenous materials) and *Source i* represents the proportion of each of these terrigenous sources affected by the erosion (the number of sources varies between 0 and x1).

2 $$[{{\rm{DNA}}}_{TERRinit}]=\mathop{\sum }\limits_{{\boldsymbol{i}}=0}^{{\boldsymbol{x}}1}(\mathop{\sum }\limits_{{\boldsymbol{j}}=0}^{{\boldsymbol{x}}2}[{{\rm{DNA}}}_{Taxaj,Sourcei}])[{\rm{Source}}\,i],$$

where [DNA_*TERRinit*_] corresponds to the sum of the concentrations of each taxa “j” (e.g. of plants) in the different terrigenous materials affected by the erosion (where the number of taxa “j” vary between 0 and x2). We hypothesise that the terrigenous materials contain different concentrations of DNA of each taxa j ([DNA_*Taxa j, Source i*_]) due to variations in; (1) spatial distribution of the taxa in the catchment, (2) DNA distribution within soil profiles, (3) soil type, and (4) biomass produced by each taxon. For instance, according to our interpretations from Lake La Thuile, the soil litter is the richest source of plant extracellular DNA (humic substances-bound DNA; Fig. 10). However, we anticipate variations in DNA content in different types of litter (for instance, forest vs meadow), due, in particular, to different biomass production levels, litter turnover, and pH conditions. The role of t hese characteristics has been proposed in a study of boreal environments, although this research assessed total DNA^45^. Data from La Thuile also suggests that organo-mineral soil horizons contain less extracellular plant DNA (clay-bound DNA) than the litter, but more than mineral (deep) soil horizons. The distribution of extracellular plant DNA in soil profiles should thus have a decreasing trend from top to bottom (Fig. 10). A lower total extracellular DNA concentration has been observed in deeper horizons (B) compared to upper horizons (A) within Inceptisols (forest soils from Mediterranean regions)^81^. In instances of buried palaeosols^82^, higher DNA content might be expected in the “palaeo” soil surface horizon. Very acidic soils and bare soils would comprise little or no extracellular plant DNA. This may, in part, explain the poor DNA record from Lake Serre de l’Homme. Moreover, glacial flour is free of extracellular plant DNA, as exemplified by the data from Muzelle.

The content of animal extracellular DNA in soil profiles can easily differ to that derived from plants. Total DNA was shown to be strongly related to animal biomass (which is much lower than the biomass of plants) as well as to soil texture, with significant leaching in sandy soils and for larger animals^41^. For the livestock, this biomass in a given space and per unit of time depends on the stock density, which is driven by animal behaviour and pastoral practices (Fig. 10). These factors will also create spatial variations in mammal DNA distribution across the catchment. However, as with plants and microbes, the highest animal DNA quantities are found in top soils^41^.

The concentration of the different sources of terrigenous materials ([Source *i*]) will depend on their erodibility (capacity to be mobilised), the slope and the connections between the sources and the lake (direct or via runoff waters and tributaries). A well-developed hydrographic web should provide terrigenous inputs from the different parts of the catchment and thus afford a more reliable reconstruction of the floristic richness at the catchment scale, as exemplified by the records of a landscape mosaic in the sediments from Lake Muzelle as well as another mountain lake, Anterne^25^. Moreover, open landscapes, with a higher erosion dynamic triggered by greater soil erodibility, should yield better spatial representativeness, such as data on the range of plants in the catchment. This process is well exemplified by the data from Lake La Thuile. However, erosion should preferentially affect the upper parts of the soils, as noted earlier. This also means that any significant developments in agricultural activities should be clearly recorded by the lake sedDNA. On the contrary, spatially extended practices, such as unmanaged grazing without stockading or animal enclosures, with less impact on the erosion dynamic, might be more difficult to detect.

Previous studies have proposed that biomass, distance from source to sink along with relief determine the terrestrial plant DNA record within sediments^2,40^. Here, our model goes further, integrating, in a more explicit manner, the mechanisms behind the production and transfer of extracellular DNA in lake sediments. In fact, our data demonstrate that the nature of erosion processes, such as sheet erosion, gully erosion, bank undercutting or glacial erosion, should be taken into consideration as they control the sources and quantities of catchment-derived extracellular DNA inputs to the lake. Furthermore, the concept of “catchment connectivity”, combining the hydrographic web and the catchment erodibility, reflects the features and processes controlling the spatial representativeness of the DNA record, which is key for high quality, reliable reconstructions, especially when landscapes possess high habitat diversity (i.e. comprise a plant metacommunity).

The second equation of the model reflects the dilution by autochthonous production (lake production):

3 $$[{{\rm{DNA}}}_{TERRSED}]=[{{\rm{DNA}}}_{TERRinit}][{{\rm{TERR}}}_{SED}]\,{\rm{or}}\,[{{\rm{DNA}}}_{TERRinit}](1\,-\,[{{\rm{AquaMat}}}_{SED}])$$

where [DNA_*TERRSED*_] is the concentration of terrestrial DNA in the sediments (log(N reads + 1)/g dry sediments), [TERR_*SED*_] is the concentration of terrigenous materials in the sediments (g of terrigenous materials/g of dry sediments) and [AquaMat_*SED*_] represents the concentration of the aquatic production.

The aquatic end-member of the sediments can include organic matter from microalgae, and aquatic plants as well as mineral matter produced or induced by aquatic organisms or chemical reactions. The dilution effect by the aquatic end-member is illustrated by the records from phases (a), (c) and (g) at Lake La Thuile and probably contributes to the poor terrestrial DNA record in lake Serre de L’Homme. In the dilution equation, we did not consider atmospheric materials as these constitute very low quantities in comparison to aquatic and terrestrial materials.

Finally, the third equation integrates the DNA degradation process within the lake water column and the sediments into the model.

4 $$[{{\rm{DNA}}}_{TERRSED}]=(1-{\rm{\alpha }})(\mathop{\sum }\limits_{{\boldsymbol{i}}=0}^{{\boldsymbol{x}}1}(\mathop{\sum }\limits_{{\boldsymbol{j}}=0}^{{\boldsymbol{x}}2}[{{\rm{DNA}}}_{TaxajSourcei}])[{\rm{Source}}\,i])[{{\rm{TERR}}}_{SED}]$$

where α is a factor of degradation (if α = 1 all the DNA is degraded and if α = 0 all the DNA is preserved). Theoretically,

$${\rm{\alpha }}=f({\rm{pH}},\,{\rm{T}}^\circ ,\,{\rm{UV}},\,{{\rm{O}}}_{2},\,{\rm{microbial}}\,{\rm{activity}},\,{\rm{salinity}},\,{\rm{sediment}}\,{\rm{composition}},\,{\rm{time}})$$

In the example of Lake La Thuile, we were able to recognise a probable negative impact of acidic conditions within the water column on DNA preservation (or on the capacity of our method to detect DNA due to the presence of humic substances). One hypothesis explaining DNA degradation in Lake Serre de l’Homme is the shallow water, favouring warm conditions, along with oxygenation. Interestingly, our data do not provide any clear evidence for a significant effect of DNA degradation over time. Indeed, the DNA concentration is not significantly higher towards the top of our cores, and all changes in DNA content occur abruptly and are always associated with sedimentological and/or geochemical changes.

Some of the factors influencing the quantity and the spatial representativeness of the DNA archived in the lake sediments are relatively constant over time (catchment slopes, lake surface to catchment surface ratio and the hydrographic web present during the Holocene). Therefore, these factors can be employed as an initial guide when choosing lakes that are suitable for the reconstruction of the catchment history (landscape and agropastoral activities). However, as the other factors could change over time (especially soil erodibility), a temporally continuous high-quality DNA record cannot be guaranteed. Therefore, the complex, temporally variable processes also require assessment. In fact, changes in the quality of the DNA record over time will limit reliable inter-period comparisons. This assessment is particularly important as the palaeosciences are primarily concerned with the identification and understanding of diachronic processes in socio-ecosystem trajectories, including tipping points and resilience. We demonstrate that the integration of data from sedimentary geology, geochemistry and soil science is a powerful approach for the assessment of potential taphonomic biases in DNA records. Similar approaches, integrating the context of sediment formation, should be more routinely adopted as interpretative tools.

The model that we propose is based on the study of only three lake-catchment systems. Therefore, a similar empirical field-study on modern sediments from a larger collection of lakes located across diverse geological and ecological environments, in order to avoid confounding variables, would be useful. Studies on a range of soils integrating the different soil horizons would also be informative and complementary. Moreover, there would be a need for experimental projects that recreate a series of different taphonomic scenarios. These projects will thus test and enhance the model proposed in the manuscript.

Lake sediment DNA is often considered as a biological/ecological proxy because it gives information about organisms. However, lake sediment DNA should also be considered as a bio-geological proxy for the following reasons; (1) the investigation and interpretation of the record must involve earth scientists who understand taphonomic approaches, and (2) it might be used to answer questions about the evolution of geological processes within a study area. Indeed, we feel that there is a potential to use the terrestrial DNA composition detected in lake sediments as a signature of the sources mobilised in a catchment in order to determine areas affected by erosion, today^83^ as well as in the past.

## Material and Methods

### Regional setting and site presentation

All three study sites are located in the French Alps, although in different ecological zones (Fig. 2A,B). The catchment of Lake La Thuile (874 m above sea level (asl)) is situated within the mountainous belt of a pre-alpine massif (the Bauges Massif, Northern French Alps). The catchment of lakes Muzelle (2105 m asl) and Serre de l’Homme (2235 m asl) are within the Ecrins massif (central French Alps), i.e. in a more internal position relative to the alpine range. These sites are at a higher altitude than Lake La Thuile. Lake Muzelle’s catchment area comprises several ecological zones/ecotones: the upper subalpine zone, the alpine zone, and the nival zone, with the presence of a relict glacier in the catchment (Fig. 2B). Serre de l’Homme is in the subalpine zone. The subalpine belt comprises the so-called “alpages” areas with high-altitude pastoral units used during the summer following the growth of grass. Given the range of altitudes covered by the sites, they cover zones that can support different types of agricultural activity. Until recently, the Lake La Thuile catchment hosted pastoral activities (including the presence of permanent farms), and multiple crops. The two other sites only support pastoral activity nowadays (Fig. 2B).

### Sites topography/geology

Each of the catchment areas studied possesses different physical characteristics (Fig. 2B). The Lake Muzelle catchment area has the highest proportion of steep slopes of the three sites, a well-developed hydrographic network, highly erodible rocks, including schist, and partial meadow vegetation, with some bare soils exposed to erosion. The lake surface constitutes <2% of the catchment, which implies there is an important “concentration effect” of sediments derived from the catchment. Combined, these characteristics lead to significant terrigenous inputs to the lake. Furthermore, the catchment comprises a glacier. Thus, a part of these terrigenous inputs is derived from glacial erosion. This type of erosion provides glacial clayey materials (“glacial flour”)^76^. At Lake La Thuile, the lake surface to catchment surface ratio is 4.7%, i.e. 2.4 times higher than for Muzelle. This implies that in Lake La Thuile, the “concentration effect” is lower than in Lake Muzelle. The slopes are also less steep, the hydrographic network is poorly developed, and the vegetation cover greater (meadows, some agricultural and forested areas) than in the catchment of Lake Muzelle. However, the presence of agricultural activities triggers significant soil erosion and thus terrigenous inputs to the lake^67,69^. The physical characteristics of Serre de l’Homme’s catchment are the opposite of those at Muzelle: high lake to catchment surface ratio (12.9%), gentle slopes, and no hydrographic network. These characteristics are not favourable for detrital supplies into the lake. However, rocks around the lake are easily erodible (sandstones), and there are some small barren/exposed areas (bare soils), which are susceptible to provide a few terrigenous (and more precisely clastic) inputs.

### Vegetation cover

Around Lake La Muzelle, the vegetation cover is dominated by subalpine and alpine meadows with herbs such as grasses (*Poaceae*), wormwood (*Artemisia*), sedges (*Cyperaceae*) and creeping willows (*Salix*)^84^. Lake Serre de l’Homme is surrounded by a eutrophic subalpine meadow with goosefoot (*Chenopodium bonus henricus*), yellow gentian (*Gentiana lutea*) and docks (*Rumex sp*.) (H. Cortot, Pers. Com.). Lake La Thuile (in a mountainous area) is surrounded by meadows and pastures. According to the exhaustive floristic survey undertaken around the lake (M. Pienne, T. Delahaye, S. Henriquet; Conservatoire Naturel de Savoie, 1999 and 2000), two types of meadows are present: a meadow with orchard grass (*Dactylis glomerata*) and heath false brome (*Brachypodium pinnatum*), which is sometimes grazed, and a mesophylic meadow dominated by grasses such as crested dogstail (*Cynosurus cristatus*), and ryegrass (*Lolium perenne*) used for grazing and mowing. Artificial grassland and kitchen garden are found in the northwest and southeast extremities of the lake. White willow (*Salix alba*), ashy willow (*Salix cinerea*), black poplar (*Populus nigra*), ash tree (*Fraxinus excelsior*) were also described at the edge of the lake. In the higher part of the catchment, there are coniferous forests comprised of spruce (*Picea abies*) on the north side, and of deciduous forest on the east side.

### Coring and dating

All lake sediment cores were taken in the deepest part of the lakes, which are located approximately in the centre of the lakes (Fig. 2). For Lake La Thuile, cores were taken using a UWITEC platform and coring devices. The sediment sequence comprises two core sites. Sections from the second core are shifted by one meter in depth in order to have overlapping sections and create a continuous sequence (THU10, N45 31.813, E6 03.394, IGSN:IEFRA00BB – IGSN codes refer to an open international database. www.geosamples.org). Cores from Lake Muzelle (MUZ12, N44 57.037, E6 05.845, IGSN: IEFRA00A4) and two from Lake Serre de l’Homme (SDH-09-P1 and P2, N44 77.459, E6 23.772, IGSN: IEFRA00AW and IEFRA00AV, respectively) were taken using a UWITEC gravity corer. Core diameters are 90 mm for La Thuile and Serre de l’Homme and 63 and 90 mm for Muzelle. Another core on Lake Serre de l’Homme (SDH-1) was also taken with a Russian corer close to the shoreline. After coring, sediment cores were stored at 4 °C.

The lake sediment cores used for DNA analyses as well as sedimentological/geochemical analyses measured 283.5 cm at Muzelle (core MUZ-12, 90 mm diameter from 0 to 130 cm depth and 63 mm from 130 to 183.5 cm depth), 549 cm at La Thuile (upper part of the core THU-10) and 81.5 cm (core SDH-09-P1) and 93 cm (core SDH-09-P2) at Serre de L’Homme. These cores cover different periods: 1700 years for Muzelle, 6450 years for La Thuile and 4000 years for Serre de L’Homme. Depending on the lakes, age-depth models are based on ^14^C dates, geomagnetic field secular variations, short-lived radionuclide measurements and known lead-pollution levels. All age-depth models were generated using the *R software* and the *R-code package ‘Clam’ version 2.2*^85^. Details about sediment lithology and the age-depth models are provided in the Supplementary Materials (Section 1.). For Lake Serre de l’Homme, several cores were used. Thus, core correlations are also presented in detail in the “sediment lithology and dating” section of the Supplementary Materials. Age-depth models were used to estimate the sedimentation rate for each lake (cm/yr).

### Sedimentological, geochemical and microfossils analyses

The cores were longitudinally cut, and a half-core was subsampled for DNA analyses (the heart of the slices, see section 4.6.1.) and for basic sedimentological analyses (edges of the slices). Samples reserved for DNA analyses were weighed wet. Edges of the sediment slices were weighed wet (Wet weight_Edge_; g) and dry (dried at 60 °C, Dry weight_Edge_; g) to determine the water content (WC) and be able to calculate the total dry weight of the sediments (Dry weight_Total_; g) and finally the total flux of sediments (Flux_*Totsed*_; g/cm^2^/yr), as follow:

5 $${{\rm{Flux}}}_{Totsed}=({\rm{Dry}}\,{{\rm{weight}}}_{{\rm{Total}}}\,\ast \,{\rm{Sedimentation}}\,{\rm{rate}})/({\rm{Half}}\,{\rm{core}}\,{\rm{surface}}\,\ast \,{\rm{Sample}}\,{\rm{thickness}})$$

Where,

$${\rm{Dry}}\,{{\rm{weight}}}_{{\rm{Total}}}={{\rm{Dryweight}}}_{{\rm{Edge}}}+{\rm{Wet}}\,{{\rm{weight}}}_{{\rm{Heart}}}\,-\,({\rm{WC}}\ast {{\rm{Wetweight}}}_{{\rm{Heart}}});$$

and

$$\,{\rm{WC}}=({\rm{Wet}}\,{{\rm{weight}}}_{{\rm{Edge}}}-{{\rm{Dryweight}}}_{{\rm{Edge}}})/{{\rm{Wetweight}}}_{{\rm{Edge}}}$$

The edge samples were then used for Loss on Ignition (LOI) analyses, except for Lake Serre de l’Homme for which the analyses were performed on another core (SDH-09-P2). Samples were firstly ground in an agate mortar, and then the standardised procedure proposed by^86^ was applied. The LOI at 550 °C and then at 950 °C burns the organic matter and carbonate particles, respectively. The contributions (%) of these two components can thus be estimated. The residue of these two successive ignitions provides an estimation of the content in non-carbonate mineral matter (%) and corresponds to alumina and silica-rich particles, i.e. clastic particles and/or biogenic silica.

In Lake Muzelle, where the sediments are dominated by the mineral terrigenous fraction, grain size measurements were also undertaken at the same sampling resolution as that employed for DNA analyses (on the other half of the core). Particle size analyses were carried out on bulk sediments using a Malvern Mastersizer S, which operates on the laser diffraction principle. Only the proportion of clays (<2 μm), will be used in this study.

Complementary information about organic matter quality is used for lakes La Thuile and Serre de L’Homme (i.e. for which sediments are the richest in organic matter). In the case of Lake La Thuile, pyrolysis Rock Eval and XRF core scanner analyses from a previous study provide indices (Hydrogen Index, HI mgHC/gTOC, Oxygen Index, OI mgO_2_/gTOC and Si/Ti as proxy of biogenic silica production) allowing us to distinguish the aquatic organic matter, the organic matter produced in the litter, the soil surface organo-mineral horizons, and the deep mineral soil horizons^67,69^. For Serre de l’Homme, the C/N atomic ratio was used as an indicator of aquatic organic matter and organic matter derived from soils and land plant macroremains^70,87^. The carbon (C) and nitrogen (N) contents were measured with an elemental analyser (CEREGE, Aix en Provence).

Pollen analyses from Lake La Thuile and spores of coprophilous fungi from Lake Muzelle were already published in^67^ and^76^, respectively. For Lake La Thuile, samples do not correspond to those used for the lake sediment DNA analyses. For Lake Muzelle, samples analysed for coprophilous fungi are the same as those for DNA.

### DNA metabarcoding approach

#### Lake sediment core sub-sampling

To avoid contamination, the sampling of the three half-cores was performed in a room dedicated to sedimentological analyses at the EDYTEM laboratory (Université Savoie Mont Blanc, Le Bourget du Lac, France), where no DNA analyses were previously performed. Sediment core slices were taken using sterilised metal plates. The edges of slices were removed using sterile scalpels as the surface of the half-core was in contact with the air, and the concave edge was in contact with water that circulates along the coring tubes. For each lake, samples were cut in two parts to perform two extractions by sediment slices. Fifty, 30 and 41 samples were taken from the cores corresponding to lakes - La Thuile, Muzelle and Serre de l’Homme, respectively. The thicknesses of sediment slices are 1 cm for lakes Muzelle and Serre de l’Homme but 0.5 or 1 cm for Lake La Thuile due to substantial variations in the sedimentation rate (greater than 10-fold variations) and thus to avoid high differences in time covered by the different samples. Sample wet weights were between 2.22 and 13.04 g for Lake La Thuile, between 4.08 and 15.63 g for Lake La Muzelle and 10.49 and 23.92 g for Lake Serre de l’Homme. These significant differences are due to different water content values, particle densities (organic vs mineral) and, in cases of lakes La Thuile and Muzelle, also due to the changes in sample thickness and core diameters, respectively. In dry weights, these differences are higher because of the wide variability of the water content, especially between the top and bottom sediments (0.58 to 9.46 g for Lake La Thuile, 1.97 to 10.88 g for Lake La Muzelle and 0.76 to 14.3 g for Lake Serre de l’Homme.

#### DNA extraction

To limit artefacts and biases that can occur in metabarcoding studies, we followed strict laboratory conditions; we performed multiple controls at the different steps of laboratory work (extraction and PCR), we analysed samples in several replicates^88^. DNA extractions were performed in the Laboratoire d’Ecologie Alpine (University Grenoble-Alpes, France), in a room dedicated to ancient DNA extraction. Eleven extraction controls were performed (3 for lakes Muzelle and La Thuile and 8 for Lake Serre de L’Homme).

DNA extraction was performed by mixing the sediment with 20 mL of saturated phosphate buffer (0.12 M Na_2_HPO_4_; pH ≈ 8) for 15 minutes. Then, the mixture was centrifuged (10 minutes at 10000 g) to recover 400 μL of the resulting supernatant. DNA was extracted from the supernatant using the NucleoSpin^®^ Soil commercial kit (Macherey-Nagel, Düren, Germany), following the manufacturer’s instructions but omitting the lysis step. The DNA extract was eluted in 100 μL of SE buffer. This method of extraction allows the retrieval of the extracellular DNA pool that is dissolved in pore water and adsorbed onto mineral surfaces. It is unlikely that organically/inorganically complexed DNA is released by DNA-desorbing phosphate buffer^58^.

#### DNA amplification and high-throughput sequencing

DNA amplification was realised in a second room of the ancient DNA laboratory using PCR. For the amplification of plants, we used the primers g-h, targeting the P6 loop region of the chloroplast trnL (UAA) intron^89^. For the amplification of mammals, we used universal primer MamP007 amplifying 60–84 bp fragment of the mitochondrial 16S gene^24^. To limit the amplification of human DNA, we used a human-specific blocking oligonucleotide (MamP007_B_Hum1, 5′-GGAGCTTTAATTTATTAATGCAAACAGTACC-C3-3′). A unique combination of 8 bp long sequence of nucleotides (tag) was added at the 5′end of each primer, in order to recognise each sample after the parallel sequencing of multiple samples^90^.

To improve the reliability of the detection/ non-detection pattern, we performed multiple PCR replicates on each DNA extract^79^. For Lake Serre de l’Homme, we performed four PCR replicates on two DNA extraction replicates, yielding eight analyses replicates. For Muzelle and La Thuile samples, we performed four PCR replicates on one single extraction replicate using the g-h and Mam-P007 primers. For mammals in the La Thuile samples, we performed 12 additional PCR replicates per sample (33 over 50 selected samples) on a second extract obtained from the same samples (which were divided into two parts).

All DNA amplifications were carried out at a final volume of 30 μL containing 2.5 μL of DNA template. The amplification mixture contained 1 U of AmpliTaq Gold^**®**^ DNA polymerase (Applied Biosystems), 15 mM Tris-HCl, 50 mM KCl, 2.5 mM MgCl_2_, 0.2 mM of each dNTP, 0.1 μM of each primer and 4.8 μg of bovine serum albumin (Roche Diagnostic). We added 2 µM of the human-specific blocking oligonucleotide to the PCR mixture in mammal analyses. For all primer pairs, the PCR mixture was denatured at 95 °C for 10 minutes, followed by 45 cycles of 30 s at 95 °C also for the denaturation, 30 s at 50 °C for the hybridation and 1 min at 72 °C for the elongation. A final elongation step was applied for 7 min at 72 °C. The PCR products were then purified and mixed (equivolume mixes) before sequencing. Seventy-two PCR controls were included for each primer.

Sequencing was carried out using the Illumina Hi-seq technology (2∗100 bp, paired-end reads), in three separate runs, one comprising four PCR replicates for plants and mammals from La Thuile and Muzelle samples; one for the additional 12 replicates of mammals in La Thuile samples and one for mammals and plants in Serre de l’Homme samples.

#### Data treatment and representation

The analysis of sequences and the taxonomic assignment were realised using the OBITOOLS software (http://www.grenoble.prabi.fr/trac/OBITools)^91^. The forward and reverse reads corresponding to the same DNA fragment were aligned and merged applying the *IlluminaPairEnd* function that takes into account the quality of merging. An “ngsfilter” file containing the list of samples and their associated combination of primer and tag was created and then used to assign each sequence to the relevant sample applying the *ngsfilter* function. Only sequences containing perfect tags and primers with a maximum of three errors were considered. The next step was to identify and merge the identical sequences for each sample using the *obiuniq* function. Afterwards, the *obigrep* function allowed the filtering of sequences based on two parameters, (1) the sequence length and (2) the sequence occurrence in the entire dataset. For plants, sequences shorter than 10 bp and sequences detected less than 100 times were removed. The same filters were applied for mammals, but we only retained sequences longer than 60 bp. *Obiclean* was then used to determine the status of each sequence in each PCR product: “head”, “internal” or “singleton”^91^. Only sequences that were more often “head” and “singleton” than “internal” in the global dataset were retained for the subsequent steps. Reference databases were built from the EMBL database with the *ecoPCR* program (gh-database-r113, mamP007-database-r113) and then used to assign a taxon to each unique sequence with the *ecoTag* function (the % of sequence similarity was calculated and specified in the final file).

For the subsequent analyses, only the sequences with a similarity >95% to taxa in the reference database were selected. We considered a sequence as present in a PCR replicate when at least five reads were counted^25^. In each lake dataset, we did not consider taxa that were only detected in one sample, or stochastically in less than two replicates (i.e. taxa always detected in only one replicate but with detections in consecutive samples were kept). To remove contaminants, we excluded taxa frequently present in extraction and PCR negative controls (in more than 5 controls, where the total number of reads was greater than 10000), and taxa allochthonous in the Alps (like *Actinidia* sp.) (see Supplementary Section 2.1 as well as Supplementary Figs 3, 6 and Table 2 for more details on contamination and on the data filtering steps). Potential impacts of the filtering procedure on the main results of the study are also presented and discussed in the Supplementary Materials (Supplementary Section 2.2. and Supplementary Figs 4 and 5).

For each PCR replicate, we summed the total number of reads corresponding to terrestrial plants, aquatic plants and mammals separately. Then, we determined the mean and standard deviation of the log-transformed total number of reads across PCR replicates, as well as the number of replicates where more than 20 reads were detected. These two parameters are positively correlated (see Supplementary Section 3), which supports the assumption that the number of reads is correlated to the DNA quantity available for amplification as suggested by previous studies on soils and lake sediments^28,45^. We normalised the log-transformed number of reads by the dry weight of sediments used for the extractions in order to obtain a proxy of the DNA concentration that we can compare with the concentrations of the main sediment components. The log-transformation helps to correct the exponential DNA amplification during the PCR. We also determined a proxy of the richness (number of MOTUs: Molecular Operational Taxonomic Units) of mammals and plants, considering the presence of the taxa (more than 5 reads). As part of this process, for terrestrial plants, the mean value and standard deviation across replicates were calculated. We also determined a “maximum richness” from the sum of reads obtained in all the replicates for each detected taxon. For Lake La Thuile, we also calculated the pollen taxon richness to compare it with the proxy of the plant DNA richness, as that had already been carried out on another lake, but with plant macroremain data^20^. For mammals, we only determined the maximum richness from the sum of reads obtained in all the replicates for each detected taxon.

## Supplementary information

supplementary materials

## Data Availability

Sequences for plant and mammal DNA (obitools output), post-filtering plant and mammal DNA datasets and sedimentological/geochemical data are deposited and available in the figshare database (10.6084/m9.figshare.9792644).
