# Supplementary material for: New insights on lake sediment DNA from the catchment: importance of taphonomic and analytical issues on the record quality
Source: Sci Rep. 2019 Oct 11;9:14676. doi: 10.1038/s41598-019-50339-1 (PMC6789010; doi:10.1038/s41598-019-50339-1)
Supplement: Supplementary file 1 — supplementary materials [file 41598_2019_50339_MOESM1_ESM.pdf]

# New insights on lake sediment DNA from the catchment: importance of taphonomic and analytical issues on the record quality

Giguet-Covex C.[1,2], Ficetola G.F.[3,4], Walsh K.[1], Poulenard J.[2], Bajard M.[2], Fouinat L.[2], Sabatier P. [2], Gielly L.[3], Messager E. [2], Develle A.L. [2], David F.[5], Taberlet P.[3], Brisset E. [6,7,8], Guiter F.[6], Sinet R. [6], Arnaud F.[2]

1- BioArch-Department of Archaeology, University of York, YO10 5DD, UK
2- EDYTEM, UMR 5204 CNRS, Univ. Savoie Mont Blanc, Pôle Montagne, 73376 Le Bourget du Lac, France
3- Univ. Grenoble Alpes, Univ. Savoie Mont Blanc, CNRS, LECA, 38000 Grenoble, France
4- Department of Environmental Science and Policy, Università degli Studi di Milano. Via Celoria 26, 20133 Milano, Italy
5- CEREGE, UMR CNRS 7330, IRD 161-Marseille Université, Technopôle de l'Arbois Méditerranée, BP 80, 13545 Aix en Provence cedex 4, France
6- Aix-Marseille Univ, Avignon Univ, CNRS, IRD, IMBE. Aix-en-Provence, France
7- Institut Català de Paleoecologia Humana i Evolució Social (IPHES). Tarragona, Spain
8- Àrea de Prehistòria, Universitat Rovira i Virgili. Tarragona, Spain

# Supplementary materials

## 1. Sediment lithology and dating

### 1.1. Lake Serre de L'Homme

Three cores from Lake Serre de l'Homme (SDH-1, SDH-09-P1, SDH-09-P2) were included in our study in order to provide an age-depth model and determine the sedimentation rate on the core we used for DNA analyses (SDH-09-P1). These sediment cores are characterised by four different units (Supplementary figure 1). Unit 1 (U1) is made of brown fibrous gyttja sediments with an organic matter content around 50-60% according to the loss on ignition at 550°C (LOI 550°C). Unit 2 (U2) is made of a brown non-fibrous gyttja with an organic matter content varying between 30 and 50%. The organic matter content varies around 30% in unit 3 (U3). The gyttja sediments from this unit are grey-brown. In unit 4 (U4), sediments have lower content in organic matter and

are mostly made of grey clays. Two $^{14}$C dates were available on cores SDH-1 and five on core SDH-09-P2 (see Supplementary table 1). Arboreal pollen/non-arboreal pollen ratio, *Abies* signal and the ruderal-anthropic herbs signal were used to project the position of the two $^{14}$C dates from the core SDH-1 on the core SDH-09-P2 (Supplementary figure 1 and table 1). Pollen on core SDH-1 (taken in 2006 with a Russian corer close to the shoreline) was analysed by S. Richer (IMBE, Université Aix-Marseille) and published in (Walsh et al., 2014). Pollen on core SDH-09-P2 (taken in the centre of the lake in 2009 with a UWITEC coring device) was analysed by R. Sinet (IMBE, Université Aix-Marseille). Positions of dates were also projected on core SDH-09-P1 based on the lithological descriptions and geochemical signals (titanium and lead/rubidium ratio) from the XRF core scanner analyses (Supplementary figure 1). SDH-09-P1 was analysed at EDYTEM (Université Savoie-Mont Blanc) using an XRF core scanner Avaatech (X-Ray beam generated with a rhodium anode and a 125 µm Beryllium window) with the following settings: run 1 at 10 kV, 1.2 mA, a counting time of 20 s and a resolution of 2 mm; run 2 at 30 kV, 0.75 mA, a counting time of 60 s and a resolution of 2 mm. SDH-09-P2 was analysed at CEREGE (Université Aix-Marseille) with a Cox Analytics *Itrax* core scanner [1]. The X-Ray beam was generated by a 3kW molybdenum tube, and the following settings were applied: 30kV, 30mA, a counting time of 20 s and a resolution of 2 mm.

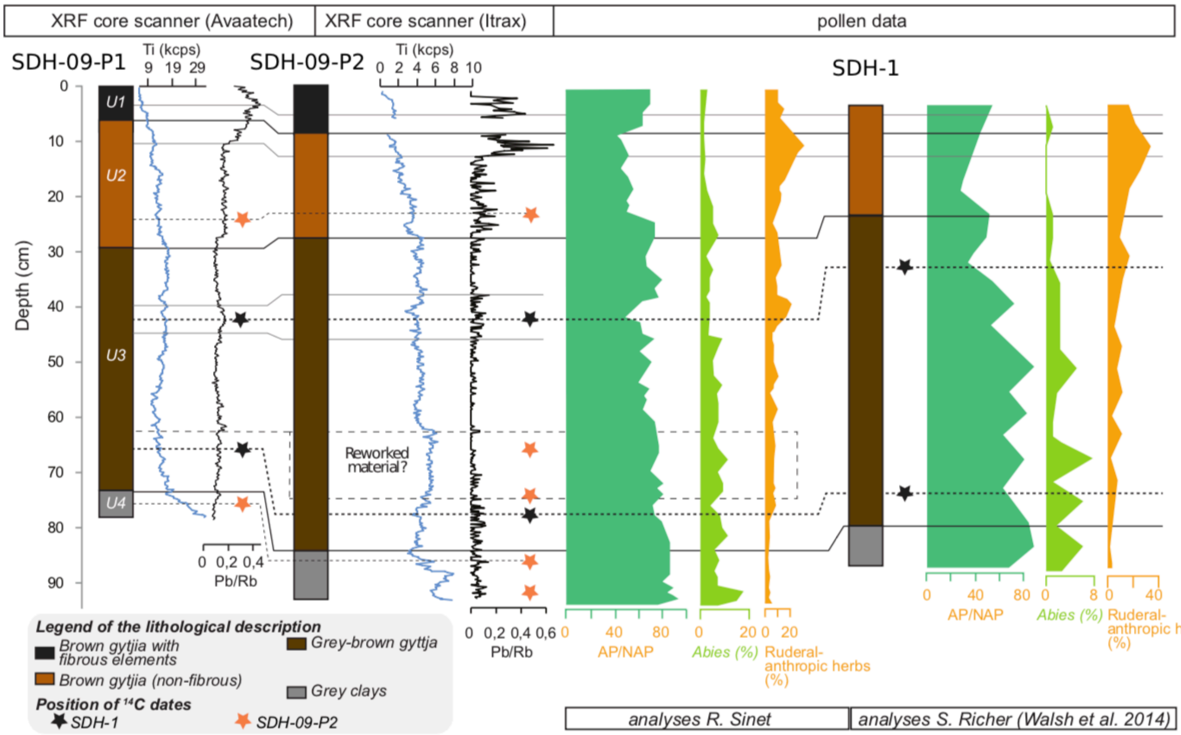


**Supplementary figure 1. Lithological descriptions and correlations for cores taken from Lake Serre de l'Homme.** Core correlations are based on titanium and lead/rubidium ratio measured by XRF core scanner for cores

SDH-09-P1 and P2, while SDH-09-P2 and SDH-1 were correlated using pollen analyses (arboreal pollen/non-arboreal pollen ratio, *Abies* and anthropogenic indicators).

Two $^{14}C$ dates from the core SDH-09-P2 were too old to respect the stratigraphic principle of superposition (Supplementary figure 2A). They were thus removed to generate the age-depth model on core SDH-09-P2. Moreover, based on the presence of these two dates from reworked materials and the enrichment in titanium only recorded in SDH-09-P2, we propose the presence of a reworked sediment deposit (or of an erosive event which mobilised old plant remains buried in soils) in the core SDH-09-P2 (Supplementary figures 1 and 2A). However, whether this deposit corresponds to reworked materials or not has no consequences for our study. For the recent period, two supplementary chronological points corresponding to recent atmospheric lead pollutions and highlighted by high Pb/Rb ratio were included to generate the age-depth model. The lead/rubidium ratio was used to highlight these lead pollution phases because it permits a correction from the enrichment effect triggered by bedrock erosion. In fact, rubidium represents a purely lithogenic element and is not affected by weathering or diagenesis processes. The upper lead peak, only recorded at 2.5 cm in the core SDH-09-P1 because of a gap in measurements on core SDH-09-P2, is attributed to the effects of the oil crisis in 1973-74 and the introduction of unleaded gasoline in 1985 [2,3]. We thus proposed 1979 $^{+}$/-6 AD as a date for this stratigraphic marker. Bellow this peak, the strong increase of Pb/Rb (at 12,5 cm for SDH-09-P2 and at 10 cm for SDH-09-P1) is attributed to the pollution triggered by the Second Industrial Revolution at the beginning of the 20$^{th}$ century and the introduction of leaded gasoline in the 1920s [2,3]. An age of 1920$^{+}$/-20 AD was proposed for this increase in Pb/Rb. The age-depth models of the two cores were generated using the *R software* and the *R-code package 'Clam' version 2.2* [4]. Models with linear interpolations were selected.

**Supplementary table 1. List of $^{14}C$ dates for Lake Serre de l'Homme.** Depth in grey and italic correspond to depth determined by correlation. Samples in bold correspond to rejected dates.

| | SDH 1 | | SDH-09-P2 | | SDH-09-P1 | | | | |
|---|---|---|---|---|---|---|---|---|---|
| | top depth (cm) | bottom depth (cm) | top depth (cm) | bottom depth (cm) | top depth (cm) | bottom depth (cm) | material dated | uncalibrated ages (BP) & uncertainty | Calibrated age ranges at 95% confidence intervals (cal. BP) |
| Poz-46475 | | | 23 | 24 | *24* | *25* | Juniperus twig | 740 +/-30 | 725-660 |
| Poz-18880 | 33 | 34 | *31* | *32* | *32* | *33* | bulk sediment | 1385 +/-30 | 1345-1275 |
| **Poz-46476** | | | **65** | **66** | | | **wood pieces (including a Brachyblast of P. cembra** | **3565 +/-35** | **3725-3970** |
| **Poz-46477** | | | **75** | **76** | | | **P.cembra and organic matter** | **3375 +/-35** | **3510-3700** |
| Poz-18881 | 73 | 74 | *78* | *79* | *65* | *66* | bulk sediment | 2930 +/-35 | 2965-3175 |
| Poz-35074 | | | 86 | | *76* | | Conifer (Larix/Picea) | 3320 +/-35 | 3465-3635 |
| Poz-46478 | | | 91 | 92 | | | pieces of wood and organic matter | 3785 +/-35 | 4005-4290 |

The age-depth models built using the $^{14}C$ dates and recent lead pollution markers are supported by older lead pollution phases detected by the XRF core scanner (Pb/Rb ratio). The most recent one starts around 900-950 cal. BP (11th century) and fits with the exploitation of two nearby mines (Faravel and L'Argentière-La Bessée) between the 10th and 13-14th centuries [5]. The other one corresponds to the Antique period (peak around 2100-2150 cal. BP, i.e. 150-200 BC). This pollution was also detected in a peat core (Fangeas) located in the neighbouring valley [5]. The origin, local vs regional/global, of this pollution could not be determined by the study by Py et al [5]. However, Roman-period pollution was also detected in several lakes and peats in French Alps [6–8] and Swiss Alps [9–11], between 2500 and 1650 cal. BP, according to the sites.

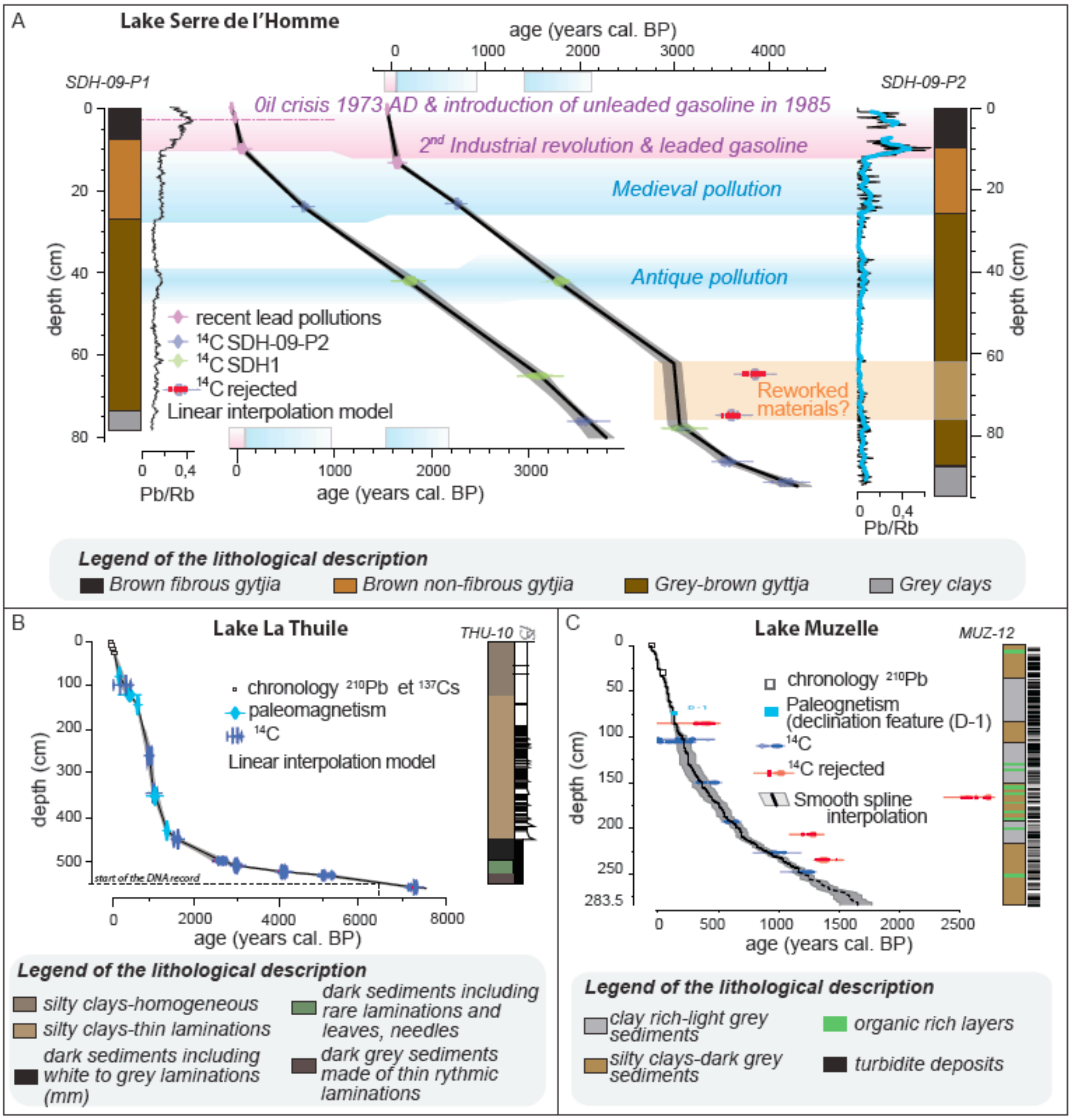


**Supplementary figure 2. Age-depth models of the three lake sediment cores.** A) Age-depth model on cores SDH-09-P1 and P2. B) Age-depth model on core THU-10. C) Age-depth model considering instantaneous deposits on core MUZ-12. The lithological descriptions of each core are also presented.

## 1.2. Lake La Thuile

The lithology of the upper 549 cm of the core from Lake La Thuile is divided into five units, which are described in detail in Bajard et al.[12] and summarized, here (Supplementary figure 2B). The two upper units represent most of the sedimentation (0-190 cm and 190-450 cm). They are mainly made of clays (10 to 40 %) and silts (40 to 80%). The lower unit is lighter and presents fine laminations, while the upper one is darker and homogeneous. The organic matter content assessed

by the LOI at 550°C is less than 10% in these two units. Between 450 and 500 cm, sediments are very dark due to higher organic-matter content (20 to 30%). White to grey laminations are visible. From 500 to 530 cm, sediments are highly enriched in organic matter (around 50 to 60%) and contain well-preserved leaves. The lower 19 cm of the core was sampled for DNA analyses and is mostly characterised by very fine rhythmic laminations alternating between bio-precipitated carbonates (mostly rhombohedral calcite crystals) and diatoms.

The age-depth model is based on nine $^{14}C$ dates and five chronological markers provided by the geomagnetic field secular variations (declination measured with three-axis 2-G enterprise cryogenic magnetometer of the CEREGE laboratory at Aix-Marseille University). The last hundred years are also constrained by short-lived radionuclide measurements ($^{210}Pb$, $^{137}Cs$, $^{241}Am$). As for Lake Serre de l'Homme, the age-depth model was generated using the *R software* and the *R-code package 'Clam' version 2.2* [4]. The linear interpolation was selected (Supplementary figure 2). More details about the sediment lithology and the age-depth model are available in Bajard et al.[12].

### 1.3. Lake Muzelle

Sediments from Lake Muzelle are characterized by the presence of turbidite deposits all along the sediment core (Supplementary figure 2C). They are interpreted as flood deposits [13]. In the continuous sedimentation, three different facies were recognised: a light grey clay-rich facies, a dark grey silty clay facies and a facies enriched in organic matter. The two first facies alternate along the core to define seven units. The organic-rich facies mostly occurs bellow 140 cm depth and in the units made of the dark grey silty clay facies (Supplementary figure 2C). The darker facies is a little enriched in organic matter relative to the lighter one (4,4% vs 3,6% according to the LOI 550°C).

As turbidite deposits represent instantaneous deposits, they were not considered (time of deposition equal to zero) in the generation of the age-depth model (Supplementary figure 2C). Ten $^{14}C$ dates were performed, but four were rejected as too old probably due to reworked materials. One chronological marker provided by the geomagnetic field secular variations (also analysed at CEREGE laboratory) was also included in the age-depth modelling using the *R software* and the *R-code package 'Clam' version 2.2* [4]. The smooth spline interpolation was preferred for this lake (Supplementary figure 2C). More information about dating and sediment lithology are provided in Fouinat et al.[13].

## 2. Filtering steps

### 2.1. Dealing with true and false presences

The summary of the numbers of reads from the high-throughput sequencing, and after the different filtering steps, is presented for plants in the supplementary figure 3. Several of these steps were used to remove potential false positives. Steps 1 to 8 (Supplementary figure 3), were realised using the OBITOOLS software (http://www.grenoble.prabi.fr/trac/OBITools)[14], which allows, in particular, the removal of potential false positives due to PCR or sequencing errors. After these bioinformatic treatments, additional filtering steps (9 to 14) were applied to remove potential sporadic contaminations. In step 9, negative controls were used to detect false positives. Unique sequences (or Molecular Operational Taxonomic Units, MOTU) detected in at least one control (>5reads) only represent 15% of the total number of MOTU assigned to 95% of similarity with sequences in the reference database but represent 65 to 80% of the read numbers (whose 10 to 16% are detected in the negative controls). On average, they mainly occur in the extraction controls for lakes Muzelle and La Thuile, and in PCR controls for Lake Serre de l'Homme (Supplementary table 2). In the filtering process, we only excluded the MOTU detected in high quantity in negative controls (in more than 5 controls with more than 10000 reads). These MOTU correspond to *Salicaceae sp.*, *Plantago sp.*, *Myriophyllum sp.*, *Asteraceae sp.* and *Lamiale sp*. for lakes Muzelle and La Thuile and *Betulaceae sp.* for Lake Serre de l'Homme (Supplementary table 2). Except for *Betulaceae sp.*, they also occur in a high number of samples with high read number and for some of them in several replicates. However, as they are detected in large quantities in the negative controls, we cannot be sure that their occurrences are not affected by contaminations. Moreover, the percentage of reads lost by the removal of these MOTU is higher in phases of lower total numbers of reads (Supplementary figure 4 and 5), which might be due to the preferential amplification of contaminants in samples containing less DNA. In Lake La Thuile, these MOTU are especially detected in very high quantity (representing up to 80% of the total read number) between 2600 and 3800 cal. BP (mostly *Lamiale sp.* and *Myriophyllum sp.*), which is a phase with a very high total number of reads but a low number of MOTU within the context of the whole dataset, i.e. without filtering (see blue area in Supplementary figure 4). Standard deviations of the total number of reads (between PCR from a common sample) are also very high, which shows a very stochastic amplification of the small number of MOTU detected in these samples and might indicate a contamination effect. The detection of a high number of DNA reads in this period is also suspicious because one extract dated to 3100 cal. BP (performed for another study) was quantified and gave a concentration lower than 0.05 ng/uL (i.e. under the detection limit of the Qubit

analysis). As presented and discussed in the manuscript, this phase is characterised by an accumulation of terrestrial plant macroremains (leaves, needles), which suggests a phase of litter erosion or direct fall of the macroremains in the lake. Humic substances present in the litter should have led to an acidification of the lake water. In addition, this hypothesis is supported by the absence of carbonate in the sediments, which only occurs during this period. Acidic conditions are not favourable to DNA preservation, which might explain the poor recording of DNA during this period. Moreover, DNA that might still eventually be present in this type of sediment is expected to be complexed with humic substances, which are not extracted by the method we used.

The next filtering step applied to limit potential false presences (step 11), was to remove MOTU detected in only one sample in each lake dataset (51 to 89 % are also detected in only one PCR). Then, MOTU only detected in one replicate per sample were discarded, unless they are detected in contiguous samples to consider the temporal autocorrelation which often affects ecological variables[15] (step 12). These two filtering steps allow the removal of MOTU considered doubtful (possibly rare taxa but also false presences). Again, the highest quantity of these MOTU is found between 2600 and 3800 cal. BP at Lake La Thuile (blue area in Supplementary figure 4). Many of the MOTU detected in low quantities in negative controls (less than 5 controls) were filtered out during these two filtering steps (21 and 26 over 49 for lakes Muzelle and La Thuile, respectively and 47 over 60 for Lake Serre de l'Homme).

The final step (13) of the filtering process common to all lakes was to remove taxa allochthonous in the Alps. In Lake Muzelle, six MOTU were assigned to *Grubbia Rosmarinifolia*, *Glycine max*, *Gaylussacia sp.*, *Styrax sp.*, *Cucumis sp.* and *Lactuca sativa*. The Grubbiaceae is a family of plants endemic to the Cape floristic region of South Africa. *Glycine max* (soy) comes from East Asia, *Gaylussacia sp.* (*Ericaceae* family) from America and the genus *Styrax sp.* from the Far East or the Mediterranean region. *Cucumis sp.* and *Lactuca sativa* are cultivated plants not supposed to grow at the altitude of the lake. In addition to some of these taxa, three other exotic taxa were removed from the Lake La Thuile dataset: *Actinidia*, *Axonopus* and *Musaceae*. *Axonopus* is a *Poaceae* from tropical and sub-tropical regions; *Actinidia* is the genus of kiwifruit and *Musaceae* the family of bananas.

In case of Lake La Thuile, a fourteenth step was applied to discard the last taxa that were detected in very high quantity only in the period when we think no or nearly no DNA was extracted (between 2600 and 3800 cal. BP). These taxa are *Pinus sp., Populus sp.*, *Frangula sp.* and

*Pooideae sp.*, which are not detected in the controls, and *Asteraceae sp.*, which is also detected in three extraction controls.

Even if they were sometimes detected in negative controls, we think that MOTU retained for the final datasets represent true signals because they possess temporal coherences, i.e. the occurrences of each plant are clustered in well-defined periods. Moreover, the temporal evolution of some of these MOTU is attested by independent methods. Specifically, pollen data (for La Thuile) and coprophilous fungi (for Muzelle) corroborated the detection of DNA from *Rumex sp.*, which was detected in 2 PCR controls (Figure 4 & 9). Likewise, for La Thuile, the DNA record of *Helianthemum nummularium* (detected in four different extraction controls with more than 5 reads) is in agreement with the pollen record (Supplementary figure 8). For Lake Serre de l'Homme, the true presence of *Potamogeton sp.* (detected in three controls) and *Sparganium sp.* (detected in four controls) is supported by the increase of the organic matter content (LOI 550°C) and the decrease in the C/N ratio (i.e. increase of aquatic organic matter relative to terrestrial organic matter) (Figure 5). Moreover, the *Potamogeton sp.* mostly occurs at the same time as the Potamogetonaceae family, which is not detected in the controls (Figure 6). However, the record of the third aquatic plant detected in Lake Serre de l'Homme (*Myriophyllum sp.)* has to be considered with caution because it is mostly detected in only one or two replicates over eight (Figure 6) and is also detected in high quantities in negative controls (Supplementary table 2). Plant community composition and ecological preferences can also be used to support the occurrence of taxa. For instance, *Pinus sp.* (detected in 2 extraction controls) can be considered as accurate detections in the case of Lake Muzelle because they are contemporaneous with *Arctostaphylos uva-ursi*, a species associated with pinewood environments (Figure 8). It is also found in association with *Rhodoreae sp.* and *Vaccinium uliginosum* (Figure 8); two species characteristic of acid environments such as *Pinus sp.* likely to be found in this region. Moreover, pollen from *Pinus sp.* (*Pinus mugo or sylvestris*) occur during the Subatlantic period (covering the last 2500 years) in pollen diagrams from a peat area just below the lake[16]. By looking in detail at our datasets, we emphasise that distinguishing between true and false presences is not straightforward.

| lake names | | Muzelle (MUZ) | La Thuile (THU) | Muzelle (MUZ) | La Thuile (THU) | Serre de l'Homme (SDH) | |
|---|---|---|---|---|---|---|---|
| number of samples | | 30 | 50 | 30 | 50 | 41 | |
| replicate number | | 4 | | | | 8 | |
| number of controls | | 64 | | | | 56 | |
| | **Data filtering steps** | **No of reads** | | **No of unique sequences** | | **No of reads** | **No of unique sequences** |
| 1 | Merging of paired-end reads | | | - | | - | - |
| 2 | Primer and tag identification | 12 401 846 | | - | | - | - |
| 3 | Dereplication | 12 401 846 | | 193 087 | | - | - |
| 4 | Filtering based on the sequence length | 12 355 683 | | 191 587 | | - | - |
| 5 | Filtering of sequences without N | 12 110 708 | | 161 773 | | - | - |
| 6 | Filtering based on the sequence occurrence (removal of sequences detected less than 100 times | 11 478 425 | | 3 224 | | - | - |
| 7 | Filtering on the status of each sequence in each PCR product (Removal of sequences more often «internal» than «head» or «singleton» | 9 723 404 | | 978 | | 9 471 448 | 2 927 |
| 8 | Assignation (percent identity ≥ 95% to database) | 7 405 225 | | 396 | | 8 700 614 | 438 |
| 9 | Removal of MOTU highly detected in negative controls | 4 910 010 | | 391 | | 8 421793 | 437 |
| 10 | Removal of «clean controls» and split of datasets for MUZ and THU | 2 445 261 | 1 714 500 | 241 | 296 | 7 781 916 | 232 |
| 11 | Removal of MOTU detected in only 1 sample in each lake dataset | 2 197 260 | 1 617 191 | 140 | 180 | 6 813 710 | 49 |
| 12 | Removal of "non-continuous detections" in less than 2 replicates | 1 850 955 | 1 544 184 | 89 | 124 | 5 916 655 | 19 (7 aquatic and 12 terrestrial MOTU but 3 different aquatic taxa and 8 different terrestrial taxa) |
| 13 | Removal of exotic MOTU | 1 836 110 | 1 475 651 | 83 | 112 | | |
| 14 | Removal of other suspicious MOTU | | 1 124 296 | | 107 (10 aquatic MOTU probably corresponding to 10 different taxa) | | |

**8. aDNA MOTU assignement (≥ 95% of similarity with taxa in the refetrence database)**

**9. aDNA MOTU absent in controls**

**List of aDNA MOTU highly detected in controls (batch Muzelle & La Thuile)**

| | scientific names | number of reads | number of PCR (>5 reads) |
|---|---|---|---|
| 1 | Lamiales | 49969 | 5 |
| 2 | Asteraceae | 32238 | 8 |
| 3 | Myriophyllum | 102399 | 9 |
| 4 | Plantago | 156669 | 12 |
| 5 | Saliceae | 120190 | 30 |

**10. aDNA MOTU in Lake Muzelle**

**«clean controls» and MOTU only detected in La Thuile**

| | Scientific names | number of reads | number of PCR (>5 reads) |
|---|---|---|---|
| 1 | Polygonoideae | 1 | 0 |
| 2 | Medicago sativa | 14 | 0 |
| 3 | Triticeae | 14 | 0 |
| | .......... | | |
| 147 | Taxus | 3877 | 19 |
| 148 | Ulmaceae | 16414 | 23 |
| 149 | Quercus | 18994 | 25 |
| 150 | Nymphaeaceae | 122372 | 57 |

**Unique sequences detected in 1 sample**

| | scientific names | number of reads | number of PCR (>5 reads) |
|---|---|---|---|
| 1 | Avena | 11 | 1 |
| 2 | Tilia | 12 | 1 |
| 3 | campanulid | 14 | 1 |
| | .......... | | |
| 98 | Trifolium | 2045 | 2 |
| 99 | Solanaceae | 15455 | 2 |
| 100 | Euphorbia | 2593 | 3 |
| 101 | Acer | 7998 | 3 |

**11. aDNA MOTU in Lake Muzelle detected in more than 1 sample**

**12. aDNA MOTU in Lake Muzelle detected at least in 2 consecutive samples**

**«non continuous detection» in less than 2 replicates**

| | Scientific names | number of reads | number of PCR (>5 reads) |
|---|---|---|---|
| 1 | Rosularia alpestris | 53 | 2 |
| 2 | Core eudicotyledons | 82 | 2 |
| 3 | Ranunculus-1 | 290 | 2 |
| | .......... | | |
| 49 | Filipendula ulmaria | 7111 | 5 |
| 50 | Ranunculus-2 | 16723 | 5 |
| 51 | Lotus | 33212 | 7 |

**13. Final list of aDNA MOTU in Lake Muzelle**

| | scientific names | number of reads | number of PCR (>5 reads) |
|---|---|---|---|
| 1 | PACMAD clade | 7884 | 2 |
| 2 | Potentilleae | 12508 | 2 |
| 3 | Pamphalea | 148 | 3 |
| | .......... | | |
| 80 | Potentilleae | 119589 | 38 |
| 81 | Asteraceae | 111445 | 49 |
| 82 | Veronica | 55462 | 51 |
| 83 | Veronica | 158657 | 54 |

**Exotic MOTU**

| | scientific names | number of reads | number of PCR (>5 reads) |
|---|---|---|---|
| 1 | Glycine max | 1113 | 4 |
| 2 | Grubbia rosmarinifolia | 1942 | 4 |
| 3 | Lactuca sativa | 3737 | 5 |
| 4 | Styrax | 124 | 5 |
| 5 | Gaylussacia | 4190 | 8 |
| 6 | Cucumis | 3836 | 9 |

**Supplementary figure 3. Information on aDNA plant datasets and flow chart of the filtering procedures.** Steps 1 to 8, were realised using the OBITOOLS software (http://www.grenoble.prabi.fr/trac/OBITools) (Boyer et al., 2016). The evolution of the number of reads and unique sequences at each step is presented, when it was calculated.

**Supplementary tables 2. List of contaminants in the sequencing run for plant DNA in lakes La Thuile and Muzelle and then in Lake Serre de l'Homme.**

**Lakes MUZELLE and La THUILE**

| | rank | scientific name | number of reads in samples | number of sample PCR >5reads | number of reads in negative extraction controls | number of negative extraction controls (>5 reads) | number of reads in negative PCR controls | number of negative PCR controls (>5 reads) | number of negative controls (>5 reads) |
|---|---|---|---|---|---|---|---|---|---|
| 1 | no rank | *campanulids* | 234 | 15 | 6 | 1 | 0 | 0 | 1 |
| 2 | tribe | *Poeae* | 4181 | 7 | 0 | 0 | 12 | 1 | 1 |
| 3 | no rank | *campanulids* | 184 | 6 | 15 | 1 | 0 | 0 | 1 |
| 4 | family | *Salicaceae* | 126 | 8 | 23 | 1 | 0 | 0 | 1 |
| 5 | family | *Salicaceae* | 427 | 18 | 23 | 1 | 2 | 0 | 1 |
| 6 | subclass | *asterids* | 394 | 18 | 31 | 1 | 0 | 0 | 1 |
| 7 | genus | *Trifolium* | 75915 | 16 | 8 | 0 | 26 | 1 | 1 |
| 8 | species | *Pentanema cernuum* | 149 | 5 | 44 | 1 | 0 | 0 | 1 |
| 9 | no rank | *campanulids* | 270 | 2 | 59 | 1 | 0 | 0 | 1 |
| 10 | species | *Vaccinium ovalifolium* | 3204 | 14 | 0 | 0 | 72 | 1 | 1 |
| 11 | order | *Asterales* | 48 | 2 | 82 | 1 | 0 | 0 | 1 |
| 12 | family | *Apiaceae* | 29 | 2 | 92 | 1 | 0 | 0 | 1 |
| 13 | genus | *Veronica* | 58945 | 65 | 145 | 1 | 21 | 0 | 1 |
| 14 | species | *Hedysarum hedysaroides* | 26537 | 9 | 354 | 1 | 4 | 0 | 1 |
| 15 | family | *Caprifoliaceae* | 2825 | 3 | 517 | 1 | 3 | 0 | 1 |
| 16 | subfamily | *Polygonoideae* | 4 | 0 | 0 | 0 | 531 | 1 | 1 |
| 17 | subfamily | *Pooideae* | 7410 | 10 | 0 | 0 | 1268 | 1 | 1 |
| 18 | family | *Betulaceae* | 82758 | 35 | 16 | 0 | 1377 | 1 | 1 |
| 19 | genus | *Equisetum* | 3999 | 12 | 1 | 0 | 1542 | 1 | 1 |
| 20 | species | *Bartsia alpina* | 8414 | 11 | 1719 | 1 | 5 | 0 | 1 |
| 21 | genus | *Gaylussacia* | 4857 | 14 | 1 | 0 | 2008 | 1 | 1 |
| 22 | species | *Pedicularis attollens* | 197 | 1 | 3195 | 1 | 0 | 0 | 1 |
| 23 | family | *Lamiaceae* | 7374 | 8 | 3908 | 1 | 4 | 0 | 1 |
| 24 | genus | *Geranium* | 10258 | 14 | 5582 | 1 | 8 | 0 | 1 |
| 25 | species | *Lomelosia brachiata* | 188 | 3 | 6317 | 1 | 0 | 0 | 1 |
| 26 | family | *Apiaceae* | 110986 | 47 | 6300 | 1 | 58 | 0 | 1 |
| 27 | family | *Ranunculaceae* | 23947 | 7 | 8060 | 1 | 10 | 0 | 1 |
| 28 | family | *Caprifoliaceae* | 1603 | 3 | 10457 | 1 | 2 | 0 | 1 |
| 29 | genus | *Caltha* | 17527 | 16 | 14973 | 1 | 8 | 0 | 1 |
| 30 | family | *Asteraceae* | 17814 | 13 | 17140 | 1 | 12 | 0 | 1 |
| 31 | species | *Medicago sativa* | 27 | 0 | 1 | 0 | 20024 | 1 | 1 |
| 32 | genus | *Veronica* | 159619 | 66 | 22709 | 1 | 40 | 0 | 1 |
| 33 | tribe | *Triticeae* | 40 | 0 | 3 | 0 | 29205 | 1 | 1 |
| 34 | genus | *Styrax* | 130 | 5 | 23 | 2 | 1 | 0 | 2 |
| 35 | family | *Apiaceae* | 186 | 8 | 76 | 2 | 0 | 0 | 2 |
| 36 | family | *Betulaceae* | 1162 | 5 | 9 | 1 | 67 | 1 | 2 |
| 37 | tribe | *Poeae* | 92096 | 11 | 11 | 0 | 5754 | 2 | 2 |
| 38 | genus | *Rumex* | 11857 | 26 | 3 | 0 | 13126 | 2 | 2 |
| 39 | family | *Betulaceae* | 97 | 0 | 21635 | 1 | 12 | 1 | 2 |
| 40 | subgenus | *Pinus* | 184831 | 19 | 12 | 0 | 71048 | 2 | 2 |
| 41 | species | *Lactuca sativa* | 51820 | 16 | 112146 | 2 | 16 | 0 | 2 |
| 42 | genus | *Alnus* | 247474 | 114 | 42 | 2 | 49 | 1 | 3 |
| 43 | genus | *Saxifraga* | 47338 | 53 | 669 | 3 | 23 | 0 | 3 |
| 44 | species | *Saxifraga oppositifolia* | 24340 | 58 | 2875 | 3 | 13 | 0 | 3 |
| 45 | genus | *Saxifraga* | 47162 | 55 | 8620 | 3 | 20 | 0 | 3 |
| 46 | species | *Saxifraga oppositifolia* | 20986 | 61 | 10168 | 3 | 13 | 0 | 3 |
| 47 | family | *Asteraceae* | 196424 | 99 | 20762 | 3 | 54 | 0 | 3 |
| 48 | family | *Betulaceae* | 90 | 2 | 23218 | 2 | 55 | 1 | 3 |
| 49 | species | *Athyrium vidalii* | 62780 | 37 | 2525 | 1 | 28189 | 2 | 3 |
| 50 | genus | *Cucumis* | 4569 | 14 | 8196 | 4 | 1 | 0 | 4 |
| 51 | family | *Betulaceae* | 17393 | 14 | 5728 | 3 | 27542 | 1 | 4 |
| 52 | species | *Helianthemum nummularium* | 113917 | 56 | 33361 | 3 | 65 | 1 | 4 |
| 53 | genus | *Picea* | 31139 | 19 | 13 | 0 | 193530 | 4 | 4 |
| 54 | order | *Lamiales* | 180033 | 102 | 24757 | 3 | 25212 | 2 | 5 |
| 55 | family | *Asteraceae* | 302264 | 117 | 32136 | 6 | 102 | 2 | 8 |
| 56 | genus | *Myriophyllum* | 326449 | 129 | 91113 | 5 | 11286 | 4 | 9 |
| 57 | genus | *Plantago* | 599174 | 127 | 13722 | 4 | 142947 | 8 | 12 |
| 58 | tribe | *Saliceae* | 624830 | 247 | 110015 | 9 | 10175 | 21 | 30 |
| | total number of PCR | | | 320 | | 16 | | 48 | 64 |

**Lake SERRE de L'HOMME**

| | rank | scientific name | number of reads in samples | number of sample PCR >5reads | number of reads in negative extraction controls | number of negative extraction controls (>5 reads) | number of reads in negative PCR controls | number of negative PCR controls (>5 reads) | number of negative controls (>5 reads) |
|---|---|---|---|---|---|---|---|---|---|
| 1 | tribe | *Poeae* | 0 | 0 | 6 | 1 | 0 | 0 | 1 |
| 2 | family | *Apiaceae* | 0 | 0 | 6 | 1 | 0 | 0 | 1 |
| 3 | genus | *Solanum* | 0 | 0 | 6 | 1 | 0 | 0 | 1 |
| 4 | tribe | *Maleae* | 1390 | 1 | 0 | 0 | 6 | 1 | 1 |
| 5 | subfamily | *Vaccinioideae* | 0 | 0 | 0 | 0 | 8 | 1 | 1 |
| 6 | species | *Grubbia rosmarinifolia* | 78 | 1 | 8 | 1 | 0 | 0 | 1 |
| 7 | family | *Salicaceae* | 0 | 0 | 9 | 1 | 0 | 0 | 1 |
| 8 | family | *Solanaceae* | 0 | 0 | 10 | 1 | 0 | 0 | 1 |
| 9 | family | *Apiaceae* | 0 | 0 | 3 | 0 | 7 | 1 | 1 |
| 10 | tribe | *Poeae* | 0 | 0 | 11 | 1 | 0 | 0 | 1 |
| 11 | no rank | *campanulids* | 0 | 0 | 11 | 1 | 0 | 0 | 1 |
| 12 | subclass | *asterids* | 41 | 1 | 11 | 1 | 0 | 0 | 1 |
| 13 | genus | *Empetrum* | 0 | 0 | 0 | 0 | 12 | 1 | 1 |
| 14 | order | *Fagales* | 45 | 2 | 8 | 1 | 4 | 0 | 1 |
| 15 | subfamily | *Solanoideae* | 0 | 0 | 13 | 1 | 0 | 0 | 1 |
| 16 | subfamily | *Asteroideae* | 1 | 0 | 17 | 1 | 0 | 0 | 1 |
| 17 | no rank | *BEP clade* | 20729 | 4 | 0 | 0 | 28 | 1 | 1 |
| 18 | genus | *Caltha* | 22228 | 4 | 25 | 1 | 4 | 0 | 1 |
| 19 | no rank | *Sanguisorbinae* | 398296 | 7 | 30 | 1 | 3 | 0 | 1 |
| 20 | genus | *Huperzia* | 0 | 0 | 0 | 0 | 36 | 1 | 1 |
| 21 | genus | *Huperzia* | 0 | 0 | 0 | 0 | 44 | 1 | 1 |
| 22 | genus | *Huperzia* | 0 | 0 | 0 | 0 | 44 | 1 | 1 |
| 23 | genus | *Rumex* | 1488 | 2 | 0 | 0 | 50 | 1 | 1 |
| 24 | genus | *Gaylussacia* | 35630 | 3 | 0 | 0 | 57 | 1 | 1 |
| 25 | order | *Asterales* | 0 | 0 | 58 | 1 | 0 | 0 | 1 |
| 26 | order | *Ericales* | 0 | 0 | 58 | 1 | 0 | 0 | 1 |
| 27 | genus | *Huperzia* | 0 | 0 | 0 | 0 | 70 | 1 | 1 |
| 28 | species | *Vaccinium ovalifolium* | 40659 | 3 | 0 | 0 | 123 | 1 | 1 |
| 29 | tribe | *Vaccinieae* | 6368 | 2 | 0 | 0 | 159 | 1 | 1 |
| 30 | genus | *Isoetes* | 24692 | 2 | 0 | 0 | 285 | 1 | 1 |
| 31 | genus | *Limonium* | 0 | 0 | 454 | 1 | 0 | 0 | 1 |
| 32 | genus | *Solanum* | 3 | 0 | 2280 | 1 | 0 | 0 | 1 |
| 33 | tribe | *Maleae* | 3155 | 1 | 0 | 0 | 2363 | 1 | 1 |
| 34 | genus | *Sparganium* | 1611392 | 35 | 30 | 0 | 2709 | 1 | 1 |
| 35 | genus | *Picea* | 152851 | 3 | 4647 | 1 | 1 | 0 | 1 |
| 36 | family | *Asteraceae* | 23608 | 1 | 4861 | 1 | 2 | 0 | 1 |
| 37 | order | *Asterales* | 5 | 0 | 6283 | 1 | 1 | 0 | 1 |
| 38 | species | *Vaccinium uliginosum* | 13 | 0 | 1 | 0 | 8332 | 1 | 1 |
| 39 | genus | *Theobroma* | 5767 | 1 | 0 | 0 | 11300 | 1 | 1 |
| 40 | genus | *Actinidia* | 74572 | 2 | 11895 | 1 | 2 | 0 | 1 |
| 41 | genus | *Avena* | 2 | 0 | 0 | 0 | 14242 | 1 | 1 |
| 42 | genus | *Glycine* | 7119 | 1 | 40250 | 1 | 2 | 0 | 1 |
| 43 | genus | *Bromus* | 9 | 0 | 45687 | 1 | 1 | 0 | 1 |
| 44 | no rank | *Sanguisorbinae* | 770 | 6 | 68980 | 1 | 1 | 0 | 1 |
| 45 | subfamily | *Pooideae* | 191706 | 4 | 0 | 0 | 77720 | 1 | 1 |
| 46 | genus | *Caltha* | 54 | 3 | 85525 | 1 | 0 | 0 | 1 |
| 47 | order | *Fagales* | 0 | 0 | 8 | 1 | 18 | 1 | 2 |
| 48 | tribe | *Poeae* | 243 | 2 | 13 | 1 | 16 | 1 | 2 |
| 49 | tribe | *Poeae* | 12640 | 2 | 67 | 1 | 109 | 1 | 2 |
| 50 | species | *Filipendula ulmaria* | 74367 | 3 | 16 | 1 | 829 | 1 | 2 |
| 51 | genus | *Empetrum* | 9361 | 3 | 2 | 0 | 3153 | 2 | 2 |
| 52 | species | *Athyrium vidalii* | 7543 | 4 | 1739 | 1 | 1658 | 1 | 2 |
| 53 | species | *Filipendula ulmaria* | 141 | 3 | 2888 | 1 | 1090 | 1 | 2 |
| 54 | order | *Lamiales* | 32992 | 3 | 4424 | 2 | 1 | 0 | 2 |
| 55 | tribe | *Saliceae* | 174359 | 5 | 6865 | 1 | 871 | 1 | 2 |
| 56 | tribe | *Poeae* | 29414 | 2 | 29261 | 1 | 23634 | 1 | 2 |
| 57 | genus | *Potamogeton* | 1942111 | 34 | 2647 | 1 | 34 | 2 | 3 |
| 58 | species | *Athyrium vidalii* | 58730 | 7 | 7460 | 1 | 8215 | 2 | 3 |
| 59 | family | *Apiaceae* | 209904 | 7 | 11363 | 3 | 11013 | 1 | 4 |
| 60 | genus | *Myriophyllum* | 961603 | 26 | 20851 | 1 | 111898 | 3 | 4 |
| 61 | family | *Betulaceae* | 38738 | 7 | 7591 | 1 | 232492 | 4 | 5 |
| | total number of PCR | | | 320 | | 32 | | 24 | 56 |

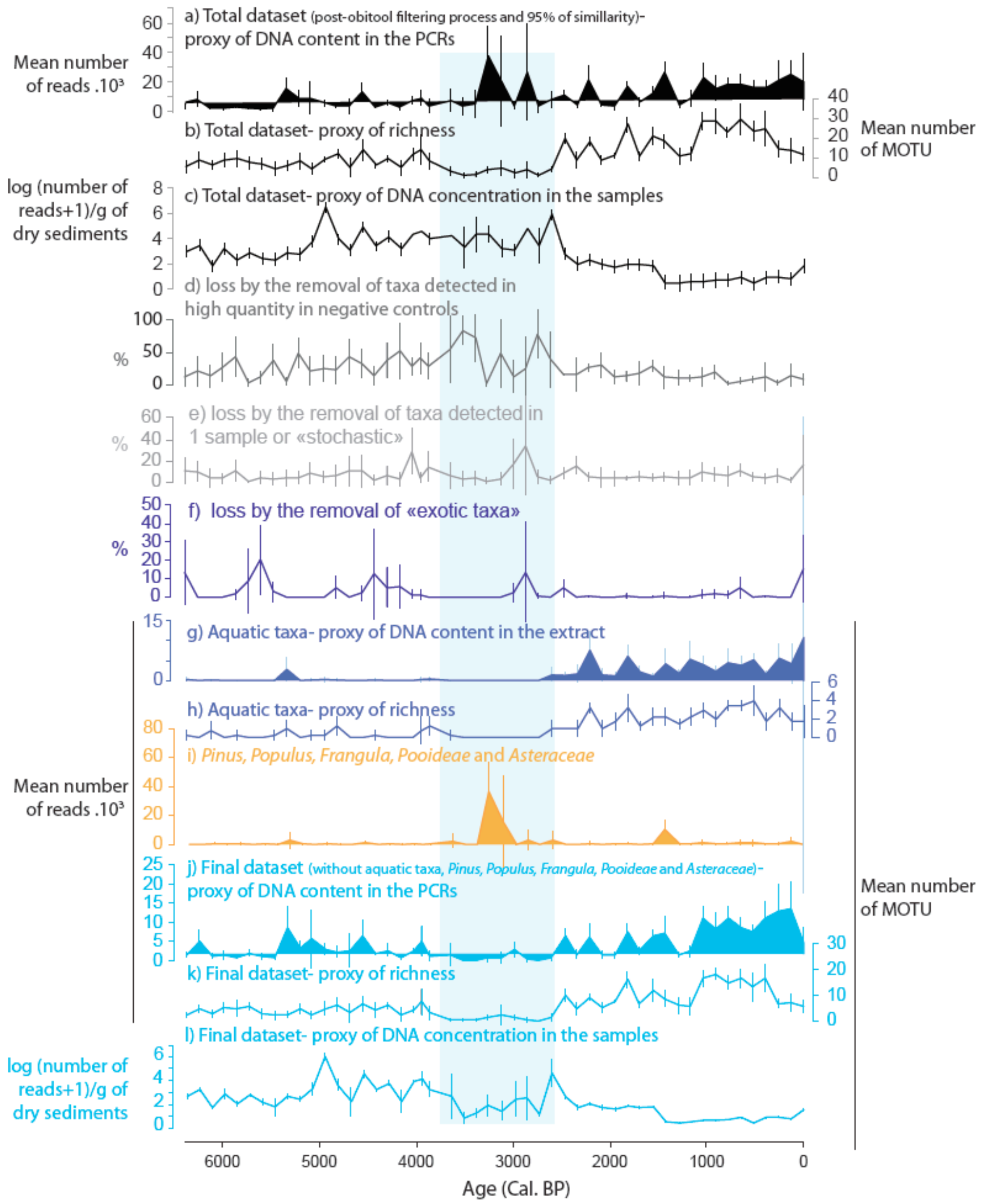


**Supplementary figure 4. Impact of the filtering process on the number of reads and of MOTU for Lake La Thuile.** Curves a, b and c represent, respectively, the number of reads, the number of MOTU and the log-transformed number of reads normalised by the dry weight of sediment (means and standard deviations) when no filtering is applied on taxa assigned at 95 % of similarity. The curves d, e and f represent the loss percentages of DNA reads at three different filtering steps: d) removal of MOTU detected in high quantity in controls, e) removal of MOTU detected in only one sample or stochastically, f) removal of exotic MOTU. The curves g and h represent the distribution over time of aquatic plants, in term of mean and standard deviation of the number of reads and of MOTU, respectively. The curve i represent the stacking of five taxa remaining after the application of the thirteenth steps of filtering and specifically detected during the period between 2600 and 3800 cal. BP (light blue area). They are removed in the fourteenth step to obtain the final curves j, k and l (mean and standard deviation of the number of reads (j) and MOTU (k) and of the log-transformed number of reads normalised by the dry weight of sediment (l)).

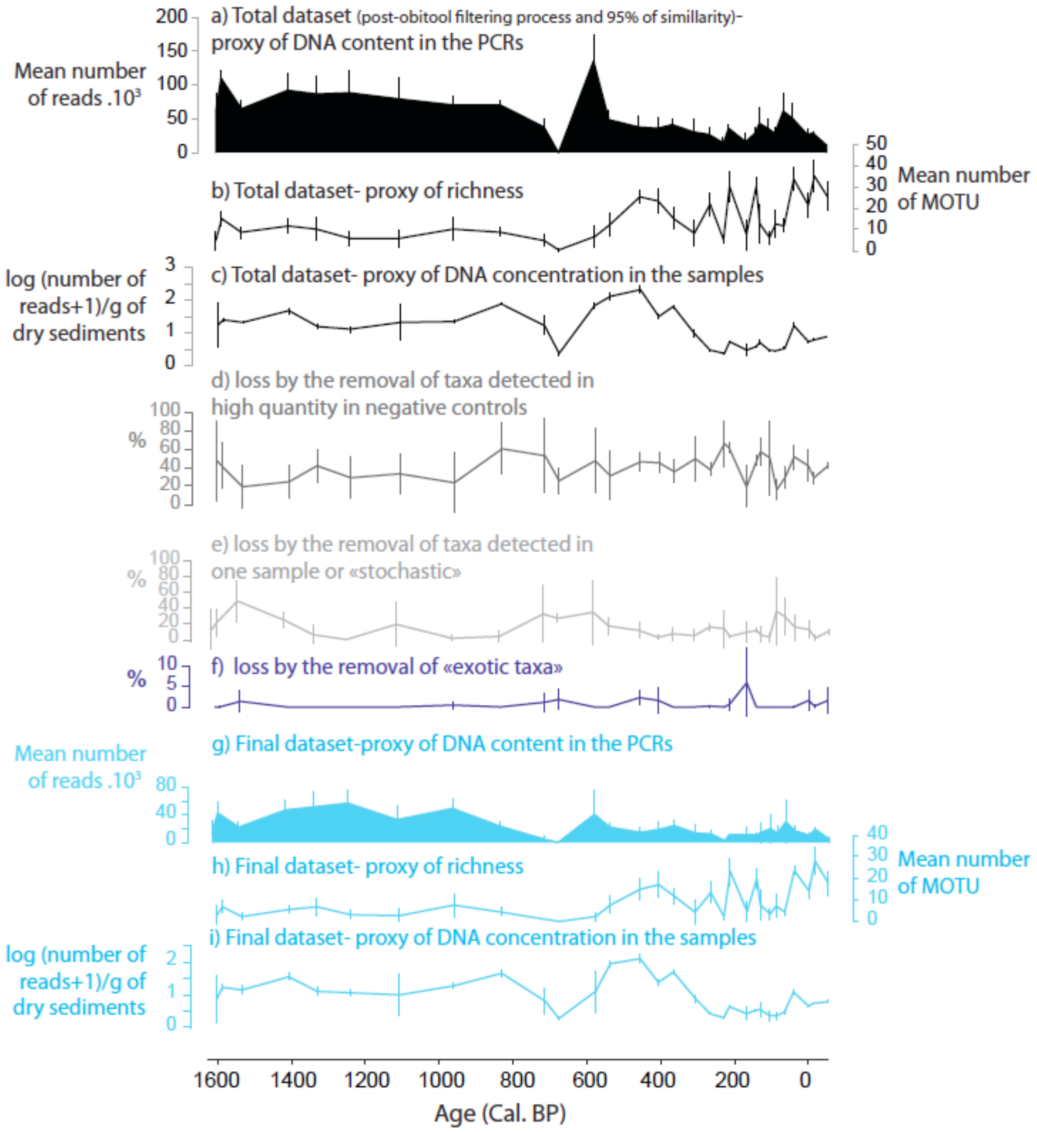


**Supplementary figure 5. Impact of the filtering process on the number of reads and of MOTU for Lake Muzelle.** Curves a, b and c represent, respectively, the number of reads, the number of MOTU and the log-transformed number of reads normalised by the dry weight of sediment (means and standard deviations) when no filtering is applied on taxa assigned at 95 % of similarity. The curves d, e and f represent the loss percentages of DNA reads at three different filtering steps: d) removal of MOTU detected in high quantity in controls, e) removal of MOTU detected in only one sample or stochastically, f) removal of exotic MOTU. After the removal of these MOTU, the final curves g, h and i are obtained (mean and standard deviation of the number of reads (g) and MOTU (h) and of the log-transformed number of reads normalised by the dry weight of sediment (i)).

For mammals, the post-obitools filtering procedure starts, as for plants, with the removal of sequences with a similarity <95% to taxa in the reference database (step 8, Supplementary figure 6). However, the next step (9) consisted in the removal of human DNA sequences because they mostly represent contaminations during sample processing. Whereas a human-specific blocking oligonucleotide was applied, sequences from *Homo sapiens* (or assigned to lower taxonomic

levels, i.e. to *Homo*, *Homininae* and *Hominoidea*) are detected in high quantity (75 to 98% of read numbers after the assignment to 95% of similarity). Then, the filtering process follows the same procedure as for plants (Supplementary figure 6). However, we removed all taxa detected in negative controls. These taxa are *Felis catus*, *Ovis aries*, *Sus sp.* and *Sus scrofa*.  Sus and Sus scrofa are common contaminants. They are mostly detected in samples from Lake La Thuile, and detections in more than one replicate are clustered between 1000 and 800 cal. BP, i.e. when other mammals (*Bos sp.* and *Ovis sp.*) are also detected (Figure 9). However, the second run performed with a higher number of replicate to increase the detection probability (12 replicates) was not able to confirm the presence of *Sus scrofa*, supporting the assumption of false presence. *Felis catus* was never detected in samples and *Ovis aries* was detected in only two PCRs in Lake Muzelle and one in Lake La Thuile.

| | *lake names* | Muzelle (MUZ) | La Thuile (THU) | Muzelle (MUZ) | La Thuile (THU) | Serre de l'Homme (SDH) | |
|---|---|---|---|---|---|---|---|
| | *number of samples* | 30 | 50 | 30 | 50 | 40 | |
| | *replicate number* | | 4 | | | 8 | |
| | *number of controls* | | 64 but 56 identified | | | 64 but 39 identified | |
| | ***Data filtering steps*** | | ***No of reads*** | | ***No of unique sequences*** | ***No of reads*** | ***No of unique sequences*** |
| *1* | *Merging of paired-end reads* | | | | - | 8 815 609 | - |
| *2* | *Primer and tag identification* | | 3 927 977 | | - | 8 178 352 | - |
| *3* | *Dereplication* | | 3 927 977 | | 188 256 | 8 178 352 | 86 500 |
| *4* | *Filtering based on the sequence length* | | 3 124 084 | | 145 782 | 7 987 849 | 75 522 |
| *5* | *Filtering of sequences without N* | | 2 687 190 | | 55 004 | 7 985 409 | 74 574 |
| *6* | *Filtering based on the sequence occurrence (removal of sequences detected less than 100 times)* | | 2 511 013 | | 853 | 7 710 767 | 1 300 |
| *7* | *Filtering on the status of each sequence in each PCR product* | | 1 940 128 | | 319 | 6 917 247 | 279 |
| *8* | *Assignation (percent identity ≥ 95% to database)* | | 1 322 537 | | 34 | 5 518 002 | 40 |
| *9* | *Removal of Homo sapiens, Homo, Homininae, Hominoidea* | | 327 649 | | 9 | 131 507 | 2 |
| *10* | *Removal of MOTU detected in negative controls* | | 62506 | | 4 | 131 507 | 2 |
| *11* | *Removal of negative controls and split of dataset for MUZ and THU* | 0 | 62463 | 0 | 4 | 131 502 | 2 |
| *12* | *Removal of unique sequences detected in only 1 sample in each lake dataset* | - | 53060 | - | 1 | 131 345 | 1 |
| *13* | *Removal of stochastic detections in less than 2 replicates* | - | 53060 | - | 1 | 0 | 0 |

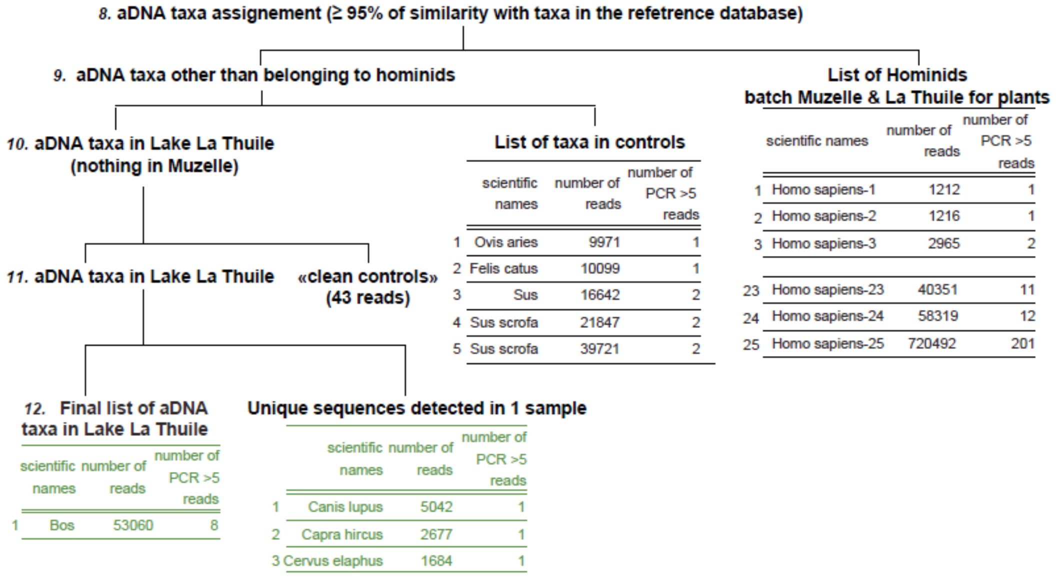

**Supplementary figure 6. Information on aDNA mammal datasets and flow chart of the filtering steps.** Steps 1 to 8, were realised using the OBITOOLS software (http://www.grenoble.prabi.fr/trac/OBITools) (Boyer et al., 2016). The evolution of the number of reads and unique sequences at each step is presented, when it was calculated. For Lake La Thuile, we only present the results of the filtering process from the first sequencing run. Another run of sequencing was performed with a higher number of PCR replicates (12) in order to increase the detection probability of mammals and attest the presence of cattle (only detected in one over four replicates in the first run). The second run improved the detection of cattle and also allowed to detect sheep (see manuscript).

## 2.2. Impact of the filtering procedure on the main results of the study

In order to assess the impact of the filtering steps (post-obitools treatment, from step 9 in supplementary figures 3) on the results of the study for terrestrial plants, the numbers of reads, of MOTU, and the log-transformed number of reads normalised by the dry weight of sediments (mean and standard deviation), are presented before and after the application of the filtering procedure for both lakes, La Thuile and Muzelle (Supplementary figures 4 and 5, respectively). The loss in percentages of DNA reads during the steps of the filtering process are also presented. The percentage of reads lost by the removal of MOTU considered as contaminants tends to be higher in phases in which lower total number of reads were detected (before the filtering step 9), except between 2600 and 3800 cal. BP at Lake La Thuile (blue area in the supplementary figure 4). As previously stated, this trend probably reflects the preferential amplification of contaminants in poor DNA samples. This hypothesis is also valid for the phase between 2600 and 3800 cal. BP at Lake La Thuile, but as probably almost no aDNA fragments were extracted, the contaminants were "over amplified" during the PCR, which leads to the very high number of reads obtained before the application of the filtering procedure. The patterns of the number of reads in the final datasets are thus only accentuated relative to the patterns before the filtering, except between 2600 and 3800 cal. BP at Lake La Thuile, when the highest numbers of DNA reads reach values close to zero after the filtering steps. Regarding the number of MOTU, the temporal patterns before and after the filtering are the same. The patterns are also retained (or slightly accentuated between 2600 and 3800 cal. BP at Lake La Thuile) for the log-transformed number of reads normalised by the dry weight of sediments, i.e. for the proxy of the DNA concentration in the samples (Supplementary figures 4 and 5). Consequently, whether a filtering procedure is applied or not, it has no impact on the conclusions of our manuscript. If we apply a more stringent filtering, i.e. removing all the taxa detected in the negative controls, it does not change the patterns either.

## 3. Metabarcoding data and DNA quantity

The goal of DNA metabarcoding is to identify taxa present in environmental samples[17]. DNA metabarcoding analyses provide numbers of reads of the different taxa detected in a set of environmental samples. Usually, this number of reads is not considered as representative of a DNA quantity but only used to determine the presence/absence of taxa. To increase the detection probability and help detect the presence of false positives, multiple replicates can be used[18,19]. The number of positive replicates has also been proposed elsewhere as a proxy for the DNA quantity in a sample (e.g. in studies of lake sediment DNA[20,21] and environmental DNA[19]). This proxy was considered as a "more conservative interpretation more appropriate for aDNA" than the number of reads[20]. However, in a previous study on environmental DNA (plant DNA) from old terraces representing a chronosequence of crop abandonments (French Alps), the number of reads was also used as a quantitative variable[22]. In fact, they showed a negative relationship between the number of reads (log(x+1)) of a specific crop plant and the number of years since the abandonment of this crop. This result was interpreted as reflecting the DNA degradation in soils, and thus implicitly means the number of reads was considered as representing a DNA quantity present in the soil. They also showed correlations between the aboveground plant biomass and the abundance in DNA reads detected in the soil. Another study on environmental DNA from lake water also showed some correlations between fish biomass and the number of fish DNA reads[23]. Regarding the lake sediment DNA, a study comparing the number of positive replicates, the number of reads, pollen data and historical maps also highlighted the potential of the number of DNA reads as a "semi-quantitative" proxy, i.e. giving a magnitude of the DNA quantity in a sample compared to another one[24]. In the present study, we also propose that the number of reads can be used as a proxy for DNA quantity, to compare between samples (analysed in the same run of sequencing). Indeed, for plants at lakes La Thuile, Muzelle and Serre de l'Homme, we found a relationship between the log-transformed mean number of reads (log(N reads+1)) and the number of replicates ($r = 0.78$, $p<0.0001$ for Muzelle; $r = 0.81$, $p<0.0001$ for La Thuile, $r = 0.74$, $p<0,0001$ for Serre de l'Homme; Supplementary figure 7). A significant positive correlation is also observed for mammals detected in Lake La Thuile ($r = 0.83$, $p<0.0001$; Supplementary figure 7). However, for mammals at Lake La Thuile and plants at Lake Serre de l'Homme, we note a wide range of read numbers when DNA is detected in only one or two replicates. This might reflect the stochasticity of DNA amplification when the amount of DNA is very low. Given the high correlation coefficient between the number of positive replicates and the mean number of reads, we propose to use the mean number of reads

between a reasonable number of replicates, even if it is probably less robust than the number of replicates and means cautions should be taken for the interpretations.

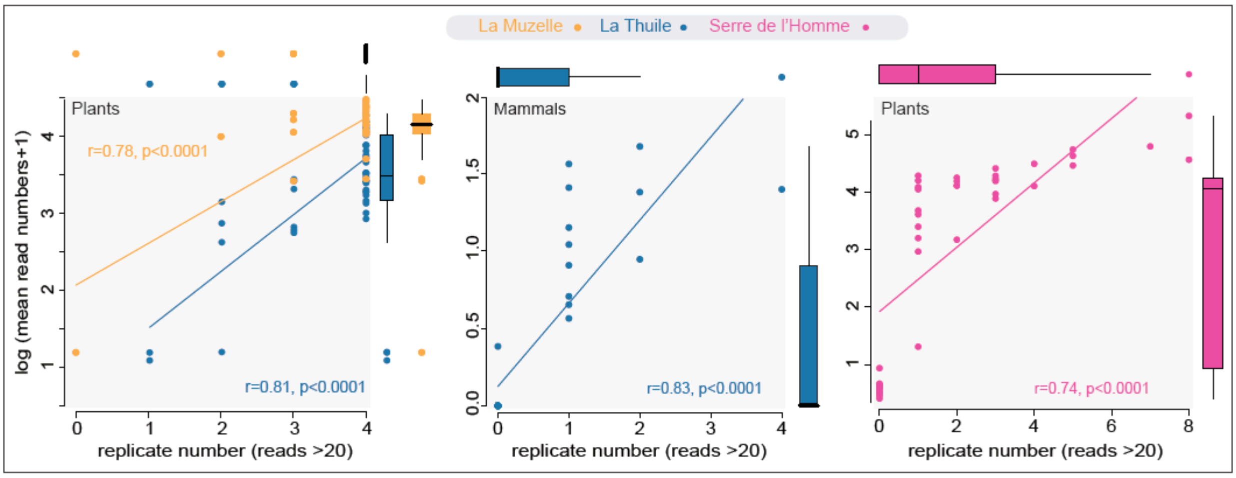


**Supplementary figure 7. Comparison between the means numbers of reads and the number of positive replicates (>20 reads).** On the left**,** bi-plot for plants in lakes La Thuile (including aquatic and terrestrial plants, 50 samples) and La Muzelle (30 samples). In the middle, bi-plot for mammals in Lake La Thuile (33 samples). On the right, bi-plot for plants in Lake Serre de l'Homme (including terrestrial and aquatic plants, 40 samples).

## 4. Complementary analysis and data on Lake La Thuile

### 4.1. PCA analysis of terrestrial plant DNA, sedimentological and geochemical data

In order to synthesise the results from the comparison of the terrestrial plant DNA concentration and richness with the sedimentological and geochemical data, we perform a PCA analysis (Supplementary Figure 8). For this purpose, we have to resample the XRF core scanner data (Si/Ti and Ca/Ti used as proxies of the aquatic production) and interpolate the data from the Rock-Eval Pyrolysis (Hydrogen and Oxygen Indexes used as proxies of a mixture between lacustrine and litter organic matters and of organic matter from deep soil horizons, respectively). This is because they were not measured at the same depths as the DNA. Despite the interpolation of some data, the PCA analysis confirms our interpretations presented in the main text. A first end-member is strongly correlated with the positive side of the first component. It includes the organic matter (LOI 550°C) mostly derived from the litter (Hydrogen Index, HI and presence of leaves in the sediments) and the DNA concentration. This end-member confirms the role of the biomass production and of the source of eroded materials in the DNA concentration. The second end-member is strongly correlated with the negative side of the first component. It represents the

detrital inputs (LOI residue and total sediment flux), i.e. the erosion and is also correlated with terrestrial plant DNA richness. This correlation can be interpreted as follows; 1) as representing the more efficient terrestrial plant DNA transfer triggered by, the higher erosion (increasing the "catchment connectivity") and/or, 2) as the result of the landscape opening which increases plant diversity. We propose that these two processes occur because the richness in the pollen data also increases; however, this increase is lower than in the DNA data. The last end-member is correlated with the positive side of the second component. This end-member represents the aquatic production (Si/Ti and Ca/Ti) and basic/neutral conditions (LOI 950°). The seven phases discussed in the main text are well discriminated by the PCA analysis. Phase (b), characterised by a high OI (Oxygen Index), provides evidence that deep soil horizons have poor DNA concentration, as the DNA concentration and OI are negatively correlated. Phase (d), for which most samples are characterised by a low DNA concentration, is negatively correlated with the second component and positively correlated with the organic matter content. This pattern probably reflects the role of acid conditions and/or of humic substances on the DNA preservation and/or our analytical capacity.

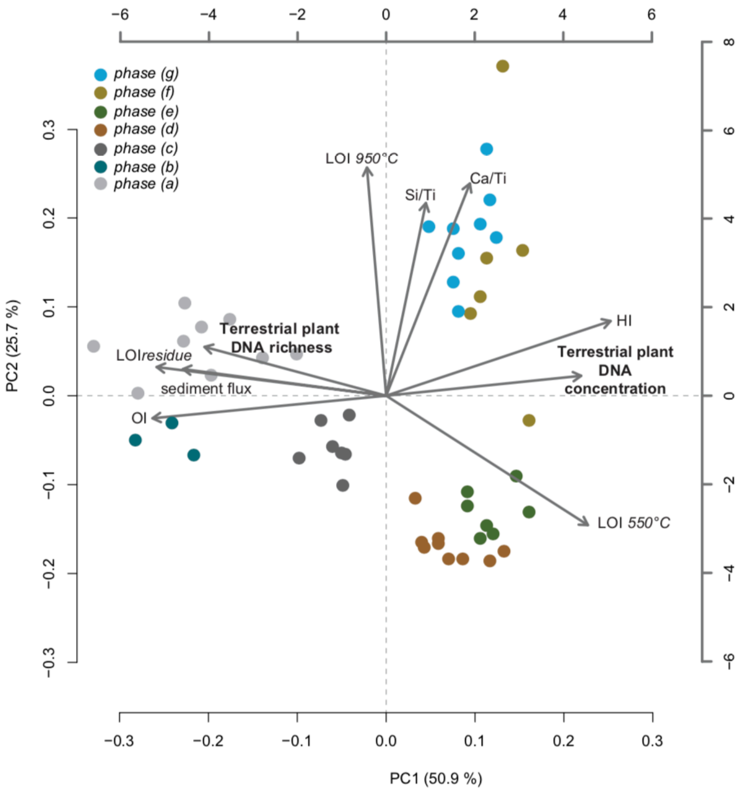


**Supplementary figure 8. PCA analysis of the terrestrial plant DNA concentration and richness and the sedimentological and geochemical data from Lake La Thuile sediments.** The first and second components represent 50.9 % and 25.7 % of the variance. HI is the Hydrogen Index (mg HC/g TOC) and OI, the Oxygen Index

(mg $O_2$/g TOC) of the organic matter. High HI values corresponds to a mixture between lacustrine and litter organic matters and high OI values show the presence of organic matter from deep soil horizons. Si/Ti and Ca/Ti are used as proxies of biogenic silica and bio-induced calcite, respectively. LOI residue and the total sediment flux are proxies of the erosion. LOI 550°C and LOI 950°C represent the contents in organic matter and carbonates, respectively. The samples from the different phases (a, b, c, d, e, f, g) used in the study (determined from the changes in the terrestrial plant DNA concentration) are presented in different colours.

### 4.2. Comparisons between DNA and pollen

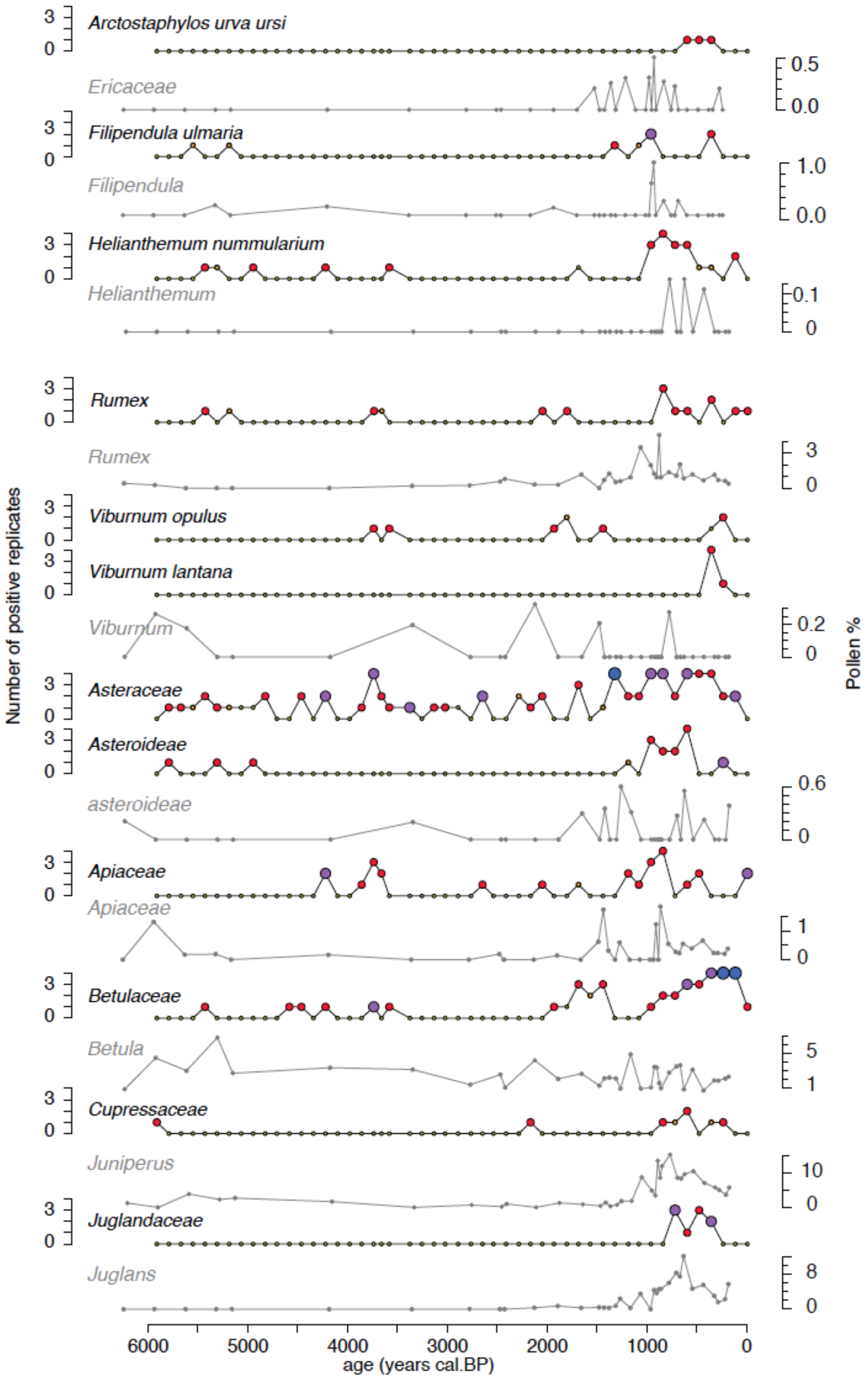


**Supplementary figures 9. Comparison between shared pollen (grey curves) and plant DNA taxa (black curves) from Lake La Thuile sediments.** Taxa possibly shared, i.e. with different taxonomic resolution like *Juglans* (pollen) and *Juglandaceae* (aDNA), are also presented.

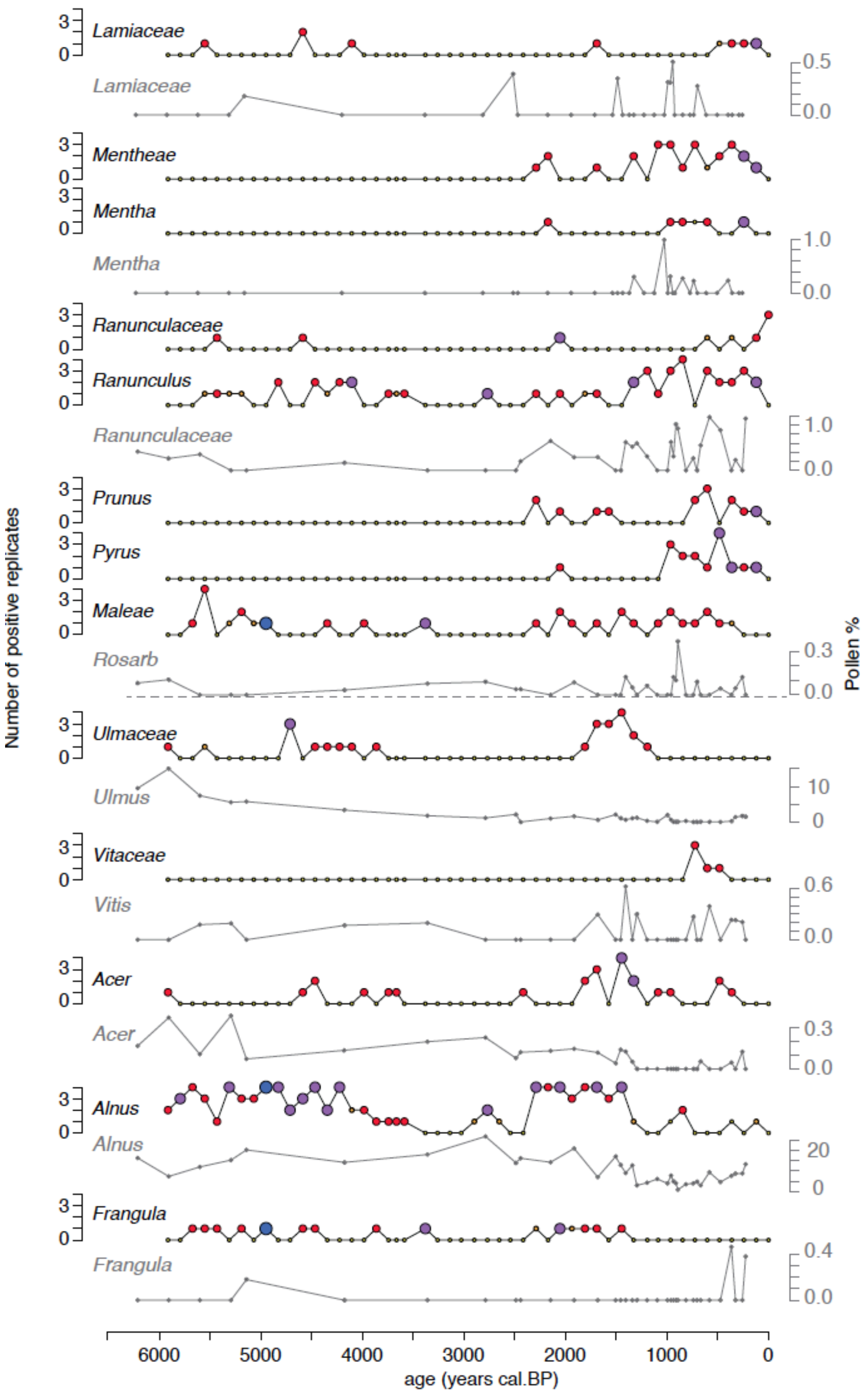


403
404 **Supplementary figures 9 (next).**
405

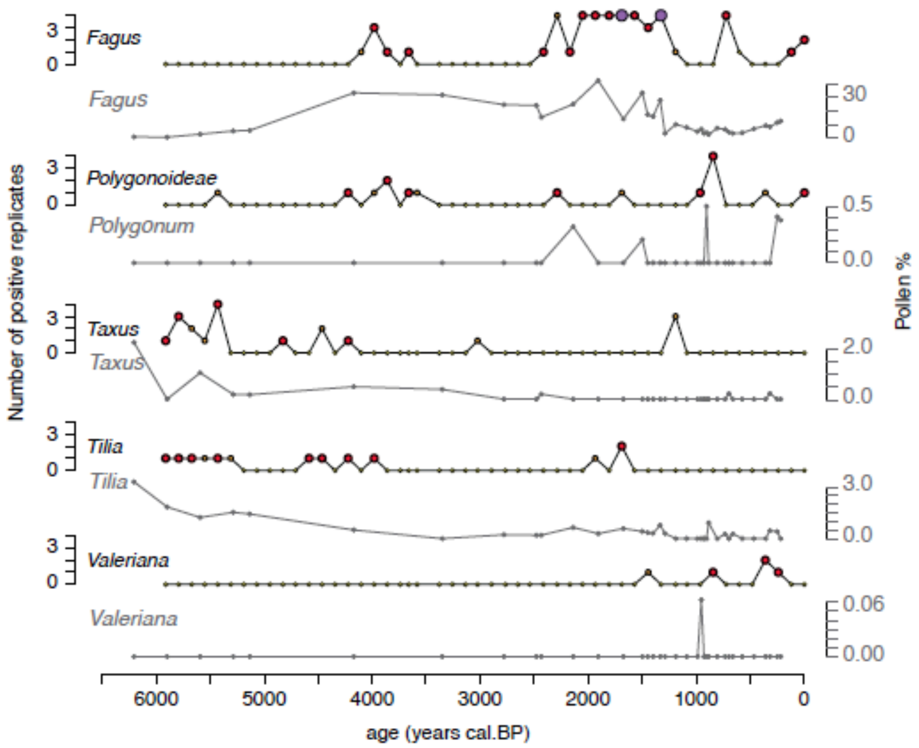


**Supplementary figures 9 (end).**

## References

1. Croudace, I. W., Rindby, A. & Rothwell, R. G. ITRAX: description and evaluation of a new multi-function X-ray core scanner. *Geol. Soc. Lond. Spec. Publ.* **267**, 51–63 (2006).
2. Arnaud, F. *et al.* A 300 year history of lead contamination in northern French Alps reconstructed from distant lake sediment records. *J Env. Monit* **6**, 448–456 (2004).
3. Weiss, D., Shotyk, W., Appleby, P. G., Kramers, J. D. & Cheburkin, A. K. Atmospheric Pb Deposition since the Industrial Revolution Recorded by Five Swiss Peat Profiles: Enrichment Factors, Fluxes, Isotopic Composition, and Sources. *Environ. Sci. Technol.* **33**, 1340–1352 (1999).
4. Blaauw, M. Methods and code for 'classical' age-modelling of radiocarbon sequences. *Quat. Geochronol.* **5**, 512–518 (2010).

5. Py, V. *et al.* Interdisciplinary characterisation and environmental imprints of mining and forestry in the upper Durance valley (France) during the Holocene. *Quat. Int.* **353**, 74–97 (2014).
6. Arnaud, F., Serralongue, J., Winiarski, T., Desmet, M. & Paterne, M. Pollution au plomb dans la Savoie antique (II–IIIe s. apr. J.-C.) en relation avec une installation métallurgique de la cité de Vienne. *Comptes Rendus Geosci.* **338**, 244–252 (2006).
7. Giguet-Covex, C. *et al.* Frequency and intensity of high-altitude floods over the last 3.5 ka in northwestern French Alps (Lake Anterne). *Quat. Res.* **77**, 12–22 (2012).
8. Guyard, H. *et al.* High-altitude varve records of abrupt environmental changes and mining activity over the last 4000 years in the Western French Alps (Lake Bramant, Grandes Rousses Massif). *Quat. Sci. Rev.* **26**, 2644–2660 (2007).
9. Shotyk, W. *et al.* History of Atmospheric Lead Deposition Since 12,370 $^{14}C$ yr BP from a Peat Bog, Jura Mountains, Switzerland. *Science* **281**, 1635 (1998).
10. Shotyk, W., Blaser, P., Grünig, A. & Cheburkin, A. K. A new approach for quantifying cumulative, anthropogenic, atmospheric lead deposition using peat cores from bogs: Pb in eight Swiss peat bog profiles. *Sci. Total Environ.* **249**, 281–295 (2000).
11. Thevenon, F., Guédron, S., Chiaradia, M., Loizeau, J.-L. & Poté, J. (Pre-) historic changes in natural and anthropogenic heavy metals deposition inferred from two contrasting Swiss Alpine lakes. *Quat. Sci. Rev.* **30**, 224–233 (2011).
12. Bajard, M. *et al.* Erosion record in Lake La Thuile sediments (Prealps, France): Evidence of montane landscape dynamics throughout the Holocene. *The Holocene* **26**, 350–364 (2016).
13. Fouinat, L. *et al.* One thousand seven hundred years of interaction between glacial activity and flood frequency in proglacial Lake Muzelle (western French Alps). *Quat. Res.* **87**, 407–422 (2017).

14. Boyer, F. *et al.* OBITOOLS:aUNIX-inspired software package for DNAmetabarcoding. *Mol. Ecol. Resour.* **16**, 176–182 (2016).

15. Chen, W. & Ficetola, G. F. Conditionally autoregressive models improve occupancy analyses of autocorrelated data: An example with environmental DNA. *Mol. Ecol. Resour.* (2018). doi:10.1111/1755-0998.12949

16. Coûteaux, M. Fluctuations glaciaires de la fin du Würm dans les Alpes françaises, établies par des analyses polliniques. *Boreas* **12**, 35–56 (1983).

17. Taberlet, P., Coissac, E., Pompanon, F., Brochmann, C. & Willerslev, E. Towards next-generation biodiversity assessment using DNA metabarcoding: NEXT-GENERATION DNA METABARCODING. *Mol. Ecol.* **21**, 2045–2050 (2012).

18. Ficetola, G. F. *et al.* Replication levels, false presences and the estimation of the presence/absence from eDNA metabarcoding data. *Mol. Ecol. Resour.* **15**, 543–556 (2015).

19. Furlan, E. M., Gleeson, D., Hardy, C. M. & Duncan, R. P. A framework for estimating the sensitivity of eDNA surveys. *Mol. Ecol. Resour.* **16**, 641–654 (2016).

20. Alsos, I. G. *et al.* Sedimentary ancient DNA from Lake Skartjørna, Svalbard: Assessing the resilience of arctic flora to Holocene climate change. *The Holocene* **26**, 627–642 (2016).

21. Pansu, J. *et al.* Reconstructing long-term human impacts on plant communities: an ecological approach based on lake sediment DNA. *Mol. Ecol.* **24**, 1485–1498 (2015).

22. Yoccoz, N. G. *et al.* DNA from soil mirrors plant taxonomic and growth form diversity: DNA FROM SOIL MIRRORS PLANT DIVERSITY. *Mol. Ecol.* **21**, 3647–3655 (2012).

23. Evans, N. T. *et al.* Quantification of mesocosm fish and amphibian species diversity via environmental DNA metabarcoding. *Mol. Ecol. Resour.* **16**, 29–41 (2016).

24. Sjögren, P. *et al.* Lake sedimentary DNA accurately records 20 $^{th}$ Century introductions of exotic conifers in Scotland. *New Phytol.* **213**, 929–941 (2017).
